# Aging and Apolipoprotein E in HIV Infection

**DOI:** 10.1007/s13365-018-0660-2

**Published:** 2018-07-09

**Authors:** Rebeca Geffin, Micheline McCarthy

**Affiliations:** Department of Neurology, University of Miami Miller School of Medicine, 1120 NW 14th St, Miami, FL 33136 USA

**Keywords:** Human immunodeficiency virus, Neurocognitive impairment, Aging, Apolipoprotein E

## Abstract

With the implementation of increasingly effective antiretroviral therapy (ART) over the past three decades, individuals infected with HIV live a much longer life. HIV infection is no longer a terminal but rather a chronic disease. However, the lifespan of infected individuals remains shorter than that of their uninfected peers. Even with ART, HIV infection may potentiate “premature” aging. Organ-associated disease and systemic syndromes that occur in treated HIV-infection are like that of older, uninfected individuals. Brain aging may manifest as structural changes or neurocognitive impairment that are beyond the chronological age. The spectrum of neurological, cognitive, and motor deficiencies, currently described as HIV-associated neurocognitive disorders (HAND), may reflect earlier onset of mechanisms common to HIV infection and aging (accelerated aging). HAND could also reflect the neurological impact of HIV infection superimposed on comorbidities linked to age and chronic inflammation, leading to a higher prevalence of neurocognitive impairment across the age span (accentuated aging). In addition, apolipoprotein E (ApoE), one of the most influential host risk factors for developing Alzheimer’s disease, has been implicated in the development of HAND. But studies differ as to whether ApoE is relevant, and whether age and ApoE interact to impair brain function in the HIV-infected patient. What is clear is that HIV-infected individuals are living longer with HIV, and therefore factors related to aging and health need to be examined in the context of current, effective ART. This review addresses the recent evidence for the influence of aging and ApoE on HIV-associated neurocognitive impairment.

**Electronic supplementary material:**

The online version of this article (10.1007/s13365-018-0660-2) contains supplementary material, which is available to authorized users.

## Introduction

In the early 1980s, HIV infection resulted in a devastating illness that led to an array of opportunistic infections and malignances, and eventually to death within a short number of years after diagnosis. With the advent of antiretroviral therapy (ART), HIV replication has been suppressed in the majority of patients to circulating viral levels that are undetectable with our current assays. HIV infection is now a chronic state with a prolonged survival since, when effective, ART has all but eliminated early mortality. According to the CDC, 45% of Americans with diagnosed HIV infection are 50 years of age or older (https://www.cdc.gov/hiv/group/age/olderamericans/index.html). By the end of 2014, this number had reached 428,724 people. It is likely that the majority of these individuals were infected at an earlier age and survived the disease for years; the new infections as recorded for 2015 by the CDC were 6725 and some of them may be newly diagnosed but not newly acquired. In fact, in 2014, 40% of people who were above the age of 55 had AIDS when diagnosed (https://www.cdc.gov/hiv/group/age/olderamericans/index.html). Recent studies predict that an infected 20-year-old with CD4+ lymphocyte levels above 350 cells/μl 1 year after starting ART may live as long as 78 years, which is similar to the life span of an uninfected individual. But this is a best case scenario and many factors are likely to reduce this predicted survival (Antiretroviral Therapy Cohort Collaboration 2017). The aging HIV-infected population now requires medical intervention for both management of HIV infection itself and management of disorders that typically accompany older age, that are HIV-associated, non-AIDS (HANA) conditions (Guaraldi et al. 2011; High et al. 2012). In the current era, ART-treated infected individuals are affected by comorbidities that are common in uninfected aging populations, including organ diseases affecting the heart (Freiberg et al. 2013; Currier et al. 2008; Triant 2012, 2014; Triant et al. 2007), the kidney and liver (Joshi et al. 2011; Joshi and Liu 2000; Kovari et al. 2013), the bones (Schouten et al. 2014), or the brain (Kaul et al. 2005) as well as non-AIDS-defining cancers (Dubrow et al. 2012), as reviewed by (Grulich et al. 2011). HANA comorbidities and systemic geriatric syndromes (multimorbidity, polypharmacy, frailty, falls) are more prevalent in HIV-infected populations than in uninfected populations of similar age. This raises the question of whether HIV infection accelerates or accentuates aging itself, as reviewed in (Pathai et al. 2014). Accelerated aging is the result of HIV infection acting through pathways and mechanisms common to aging. For example, age-related immunological senescence, typical of the elderly, is also manifested in the HIV-infected population (Appay and Sauce 2017), driven by immuno-inflammatory mechanisms that are similar in HIV infection and aging (reviewed in Bhatia et al. 2012). Accentuated aging is the result of HIV infection acting as an additional risk factor for chronic disorders associated with aging; thus, the prevalence of the disorder is increased in the HIV-infected population across the age span. Pathai et al. propose that HIV-related acceleration versus accentuation of aging is probably organ and disease/condition specific. Immune system changes more clearly reflect accelerated aging (e.g., immune senescence). Specific end-organ diseases during HIV infection present a more complex picture, but several suggest accentuated aging, e.g., cardiovascular disease, diabetes mellitus, or bone fractures (Pathai et al. 2014). Given that nervous system aging is impacted by intrinsic changes as well as other organ pathologies (e.g., cardiovascular), HIV infection can be expected to potentiate both accelerated and accentuated neurological aging.

Neurocognitive impairment (NCI) is among the persistent, even growing clinical challenges associated with aging in the treated HIV-infected population (McArthur and Brew 2010; McArthur et al. 2010). NCI persists in ART-treated populations (Heaton et al. 2011; Sacktor et al. 2016; Simioni et al. 2010), and is likely the clinical manifestation of underlying cellular mechanisms driving HIV neuropathogenesis despite suppression of circulating viral load. As early as 8 days after the suspected time of infection (Valcour et al. 2012), HIV can be detected in the cerebrospinal fluid (CSF) and rapidly enters the brain within 15 days after infection (Davis et al. 1992). Brain invasion presumably results from a Trojan horse mechanism like that initially proposed to describe neuroinvasion by Visna virus after infection of peripheral monocytes (Peluso et al. 1985). HIV-infected monocytes reach the brain across the blood–brain barrier and differentiate into macrophages, which increases virus replication dramatically. Macrophages, together with microglia and to some extent astrocytes, constitute the principal population of cells supporting HIV replication in the brain (Liu et al. 2004; Thompson et al. 2011) and drive the neuropathogenesis of neurological disease. Potential mechanisms of neuropathogenesis include direct neurotoxicity by the HIV proteins Tat (Buscemi et al. 2007; Fan and He 2016; Fields et al. 2015; Rahimian and He 2016; Sabatier et al. 1991) and gp120 (Bachis et al. 2006; Bachis et al. 2012; Mocchetti et al. 2012; Nosheny et al. 2006; Reddy et al. 2012; Wenzel et al. 2017; Zhou et al. 2017), and the pro-inflammatory milieu generated by factors released by infected and/or immune-activated microglia and astrocytes (Faissner et al. 2014; Wu et al. 2015; Zenon et al. 2015). HIV replication in the central nervous system (CNS) is clinically associated with a spectrum of neurological abnormalities collectively known as HAND (HIV-associated neurological disorders) and subdivided into asymptomatic neurocognitive impairment (ANI), minor neurocognitive disorder (MND), and HIV-associated dementia (HAD) (reviewed by Wendelken and Valcour 2012). The severe neurological deficits of HAD were more prevalent before the advent of ART. Numerous reports have chronicled these severe neurological deficits acquired by infected individuals in the 1980s and 1990s. But with the advent of ART, the spectrum of neurological disease has shifted over the past two decades towards a more chronic NCI encompassed by ANI and MND (McArthur et al. 2010), and that has similarities to cognitive impairments seen in uninfected aging populations that are affected by host genetic factors (e.g., apolipoprotein E) and acquired organ-related or systemic conditions (e.g., vascular disease). Accordingly, this review will focus primarily on neurological disorders described over the past two decades in studies of the ART-treated populations living and aging with HIV infection.

## Neurocognitive changes and chronic HIV infection: accelerated or accentuated aging

There remains debate on the relationship between aging and HIV-associated neurocognitive impairment (Bhatia et al. 2012; Pathai et al. 2013; Sheppard et al. 2017). But numerous studies over the past two and a half decades, conducted with relatively large HIV-infected cohorts and age-matched seronegative (SN) controls, overall suggest that both accelerated and accentuated aging may affect the neurological course of HIV infection (Table 1).

**Table 1:** Studies on neurological manifestations of HIV and aging

Publication	Study population	Measures	Findings
Valcour et al. (2004a)	Hawaii Aging with HIV Cohort (HAHC) HIV+ *n* = 202 > 50 years *n* = 106 67% on ART < 50 years *n* = 96 78% on ART	Longitudinal cohort Neuropsychological tests	Twofold risk for HAD associated with age < 50 years
Becker et al. (2004)	HIV+ *n* = 290 ^a^age 34.3 years 17% on ART SN *n* = 124 age 38.3 years	Neuropsychological tests	Neurocognitive disorders more prevalent in the HIV+ group older than 50 years than in the younger group. HIV viral load at entry was higher in individuals who later developed cognitive dysfunction
Chang et al. (2004)	HIV+ *n* = 100 61 with AIDS dementia complex Asymptomatic = 39 7% naïve to ART 93% on ART SN *n* = 37 Stratified for age > 40 years (overall median)	MRS: glial (myoinositol, MI to creatine, Cr) and neuronal (*N*-acetyl compound, NA, to creatine, CR) metabolites	HIV+ had higher glial metabolites in white matter Elevated MI in white matter is early in HIV disease, before cognitive decline Dementia is associated with lower neuronal marker in frontal white matter HIV infection and aging had additive effects on glial metabolites in basal ganglia and white matter Lower NA in frontal white matter of younger HIV+ (< 40 years); this may reflect greater inflammatory response Elevated glial metabolite (MI) persisted across the age span
Sacktor et al. (2007)	HIV+ *n* = 254 > 50 years *n* = 133 80% on ART 20–39 years *n* = 121 67% on ART	Neuropsychological tests -Verbal and visual memory -Verbal fluency -Psychomotor speed	Age associated with poorer performance in tests and with dementia
Chang et al. (2008)	HIV+ on HAART *n* = 39 age 47.4 years SN *n* = 32 age 46.7 years	Neuropsychological tests DTI measured at entry and after 1 year	HIV+ had higher mean diffusivity of genu of corpus callosum compared to SN after 1 year This was associated with lower cognitive performance
Woods et al. (2010)	HIV+ ≤ 40 years *n* = 35 71.4% on ART SN ≤ 40 years *n* = 20 HIV+ ≥ 50 years *n* = 48 75% on ART SN ≥ 50 years *n* = 15	Prospective memory tests	Additive effects of aging and HIV infection on event-based prospective memory. The poorest performers were HIV-infected, older individuals
Valcour et al. (2011)	Hawaii Aging with HIV Cohort (HAHC) HIV+ > 50 years old *n* = 136 73% on ART HIV+ < 40 years old *n* = 110 75% on ART SN > 50 years old *n* = 106 SN < 40 years old *n* = 98	Neuropsychological tests	No correlation between age and neuropsychological performance
Scott et al. (2011)	HIV+ ≤ 40 years old *n* = 24 66.7% on ART SN ≤ 40 years old *n* = 24 HIV+ ≥ 50 years old *n* = 48 75% on ART SN ≥ 50 years old *n* = 20	Neuropsychological testing -Episodic learning and memory -Executive functions -Visuoconstruction	All three measures were affected by aging HIV infection affected verbal learning and memory
Ances et al. (2012)	HIV+ on HAART *n* = 26 age 40 years HIV+ HAART naïve *n* = 26 age 34 years SN *n* = 26 age 34 years	Neuropsychological tests -Memory -Psychomotor speed -Executive function Neuroimaging: Brain volume	HIV+ performed worse in neurological tests than SN HIV+ had reduced brain volumes in some but not all brain regions compared to SN HAART did not affect these measures HIV and age were independent correlates of lower brain volumes
Sheppard et al. (2015)	Neurocognitively normal *n* = 146, tested at entry and 14 months HIV+ ≤ 40 years *n* = 27 85% on ART SN ≤ 40 years *n* = 24 HIV+ ≥ 50 years *n* = 56 89% on ART SN ≥ 50 years *n* = 39	Neuropsychological tests	HIV+ had a 5-fold risk of developing a neurocognitive disorder over approximately 1 year Age not a correlate of developing neurocognitive deficiencies
Marquine et al. (2014, 2016)	Veterans Aging Cohort Study (VACS) Longitudinal study (up to 6 years follow-up) HIV+ *n* = 655, age 42.5 years 61% on ART *n* = 392 with no baseline impairment	VACS Index (composite marker of disease in HIV+, marker of all-cause mortality) Neurocognitive testing	VACS Index associated with risk for worse global neurocognitive performance (2014) With time, increasing VACS Index was associated with higher risk of developing neurocognitive impairment in individuals who had normal tests at baseline (2016)
Avci et al. (2016)	Younger *n* = 125 HIV+ ≤40 years *n* = 77 84% on ART SN ≤ 40 years *n* = 48 Older *n* = 189 HIV+ ≥ 50 years *n* = 112 90.2% on ART SN ≥ 50 years *n* = 77	Tests of prospective memory (PM) -Memory for Intentions Screening Test (MIST) -Naturalistic PM task -Prospective and retrospective memory questionnaire (PRMQ)	Effects of aging vary by cue type and delay interval on the MIST Effect of HIV on the MIST varies per cue type and delay interval HIV disease interacts with age on naturalistic PM task -HIV+ older perform equal to HIV+ younger and SN younger -SN older perform better than others
Cole et al. (2017)	HIV+ *n* = 162 All on ART SN *n* = 105 Ages 45–80 years	Brain-PAD (brain predicted age difference score) based on MR neuroimaging Cognitive testing	Brain-PAD score higher in HIV+ Higher brain-PAD score associated with poorer cognitive performance PAD score not associated with age, duration of infection
Sheppard et al. (2017)	HIV+ 50–65 years *n* = 40 All on ART SN 50–65 years *n* = 48 SN > 65 years *n* = 40 (“older old”)	Neuropsychological tests -Tests of attention	HIV-infected individuals performed more poorly than the age-matched SN but similar to much older SN controls with respect to auditory attention Did not observe similar changes in other neurocognitive domains (episodic memory, language, or executive functions)
Strain et al. (2017)	HIV+ *n* = 92 age 49.1 years SN *n* = 66 age 38.6 years	Diffusion basis spectral imaging (DBSI): cellularity Neuropsychological (NP) testing for: Memory Psychomotor speed Executive function	Diffuse increases in cellularity in brains of HIV+ compared to SN Cellularity declined with age in HIV+, but not significant when corrected for sex, duration of ART No difference in NP test performance between HIV+ and SN

*ART* antiretroviral treatment

^a^Age values are mean ages unless otherwise specified as median or age range

Neuropsychological studies dating back to the previous decade did indicate that age is a risk factor for HIV-associated NCI (Sacktor et al. 2007; Valcour et al. 2004a). Valcour et al. (Valcour et al. 2011; Valcour et al. 2004a; Valcour et al. 2004b) studied the Hawaii Aging with HIV-1 Cohort (HAHC), a longitudinal, prospective cohort that has included hundreds of subjects, including those older than 50 years of age and younger than 40 years, with carefully matched SN controls. Their earlier study (Valcour et al. 2004a) that did not include SN individuals used neuropsychology testing and neurobehavioral data to designate diagnoses by a consensus panel that included neurologists and neuropsychologists. The study identified a twofold increased risk for HAD in patients greater than 50 years of age. In a follow-up report published 7 years later and using a slightly larger cohort of HIV-infected individuals from the same HAHC cohort with age-matched SN controls, there was limited evidence for any correlation between age and neuropsychological performance (Valcour et al. 2011). The differences in results between the two studies were attributed to the use in the latter study of models that included interaction variables and means and frequency of poor performance on neuropsychological tests, instead of cutoff points defining neurological impairment. In addition, a carefully selected control group of matched SN individuals helped normalize the data for comorbidities that are found in the aged population independently of HIV infection. Sacktor et al. (2007) compared neuropsychological test performance between 133 older (≥ 50 years) versus 121 younger (20–39 years) HIV-infected individuals, with or without NCI or dementia. They found that age was associated with lower performance in domains including memory, executive functions, and fine motor skills. It was unclear whether the differences were the result of age or age-associated comorbidities since the study did not include an age-matched HIV SN population. Using the Veterans Aging Cohort Study (VACS), Marquine et al. (Marquine, Montoya et al., 2016; Marquine, Umlauf et al. 2014) examined the risk for global NCI in HIV-infected individuals that had been scored by the VACS Index, a risk index that combines age, HIV biomarkers (e.g., viral load and CD4 count), and non-HIV factors that take into account HANA. The VACS Index had been validated as a marker of all-cause mortality in this cohort. Using 601 participants (mean age 41.9 years, overall range 18–76 years, age 50–64 years *n* = 95, age ≥ 65 years *n* = 13), the VACS Index was associated with risk for concurrent global NCI (Marquine et al. 2014), particularly with older age. In a longitudinal study following 655 HIV-infected participants (mean age 42.5 years, range 18–70 years) for up to 6 years, baseline VACS Index scores were not significantly associated with incident NCI in demographics-adjusted analyses. But time-dependent analyses showed that increases in VACS Index scores were significantly associated with worse global and domain neurocognitive performance and higher risk of developing neurocognitive deficits among patients who had normal neurocognitive tests at baseline (Marquine et al. 2016). While the VACS cohort and the VACS index provided a well-characterized study population and risk marker (VACS Index) for longitudinal study of NCI in HIV infection, the cohort was still relatively young, with mean age < 50 years of age.

These earlier studies highlighted the need for a carefully matched SN control population in studies where neurocognitive performance may be associated with aging but may also be impacted by other health and lifestyle factors such as alcohol or tobacco use. More recent neuropsychological studies, reported in the current decade, suggest that age and HIV infection have an additive impact on neurocognitive performance, with younger HIV-infected individuals performing comparably to older SN individuals in neurocognitive tests, suggestive of accelerated aging (Avci et al. 2016; Scott et al. 2011; Woods et al. 2010). Sheppard et al. (2015) started with a group of cognitively normal individuals and tested their neurocognitive performance approximately 14 months later. Younger individuals were < 40 years of age, and older were > 50 years of age. In a study population of 146, 63 were SN. This study found that HIV seropositivity was a correlate of developing neurocognitive deficiencies in follow-up tests, but age was not. Interestingly, SN individuals with hepatitis C virus (HCV) infection were more likely to develop neurological abnormalities, but HCV infection was not a factor in the HIV-seropositive group. Sheppard et al. (2017) subsequently investigated accelerated neurocognitive aging by testing older HIV-infected individuals (aged 50–65 years) against age-matched SN and much older (“older old,” ≥ 65 years) SN controls. Using clinical measures of attention, the investigators found that the HIV-infected group performed more poorly than the age-matched SN but were similar to the much older SN controls with respect to auditory attention. The differences suggested both accentuated and accelerated aging. However, the investigators did not observe similar changes in other neurocognitive domains, including episodic memory, language, or executive functions.

In addition to neurocognitive tests, brain imaging methods have been used to correlate brain structure or metabolism with cognitive function in the aging HIV-infected population (reviewed in Holt et al. 2012). Magnetic resonance (MR)-based structural studies have shown brain cortical atrophy and/or subcortical regional volumetric loss in HIV-infected individuals, volume loss that is greater than age-related and independent of HAND status. Normal aging is an independent factor contributing to brain atrophy in the frontal and temporal lobe gray matter and white matter. But HIV infection and aging may independently contribute to regional brain volume loss, particularly in subcortical regions, even with ART-managed infection (Ances et al. 2012; Chang et al. 2011), as reviewed in Holt et al. (2012). In HIV-infected subjects, specific subcortical brain regions, e.g., the putamen, have shown volume loss across the age span (Chang et al. 2011), which Chang termed a “legacy effect” (Chang 2018). While early and persistent brain volume loss during HIV infection could reflect accentuated aging, it may also be the result of the “legacy” of accumulated disease effects such as chronic neural inflammation and oxidative stress over time. Alternatively, other subcortical regions, e.g., the corpus callosum, have shown increased volume loss with age, suggesting accelerated aging. MR diffusion tensor imaging (DTI) detected increased mean diffusivity of the genu of the corpus callosum over 1 year in a cohort of 39 clinically and cognitively stable HIV-infected subjects (mean age 47.4 years) compared to 32 SN controls (mean age 46.7 years), suggesting greater than age-related inflammatory changes occurred in that region (Chang et al. 2008). In a subsequent study, Strain et al. (2017) used diffusion basis spectral imaging (DBSI)-derived cellularity as a measure of restricted water diffusion associated with inflammation in the brain. Their study cohort included 92 virologically suppressed HIV-infected subjects on stable ART regimens (mean age 49.1 years) and 66 SN controls (mean age 38.6 years). Diffuse increases in cellularity were present in the brains of HIV-infected individuals compared to SN control subjects. Similar diffuse increases in cellularity were seen in an age-sex matched subset of 40 HIV-infected and 40 SN control subjects. Considering all study subjects, cellularity declined with age in the HIV-infected group but not the SN controls. However, after adjustment for sex and duration of ART (mean 10.3 ± 7.1 years), this inverse relationship between cellularity and age was not significant. Neuropsychology test performance was not significantly different between HIV-infected versus SN controls tested for memory, psychomotor speed, or executive function. Strain et al. concluded that DBSI-derived cellularity is a particularly sensitive brain imaging measure, revealing diffuse inflammation in HIV-infected subjects regardless of cognitive impairment and even with effective viral suppression by ART. In a study of 48 older (age > 50 years) HIV-infected subjects and 25 SN controls, Sacktor (2018) measured brain amyloid deposition by positron emission tomography (PET) using the [18F] AV-45 tracer. The subjects were stratified by (1) serostatus, (2) HAND status (HIV-infected only), and (3) decade of life. Comparing the HIV-infected to the SN, there was significant increased global and regional tracer uptake only in the HIV subjects aged 50–59 compared to SN aged 50–59. No significant differences were detected between HIV-infected and SN subjects aged 60–69. There were no differences in median global or regional uptake when HIV-infected subjects were stratified by HAND stage. Neuropsychological testing did not find any correlation between cognitive impairment and the higher amyloid deposition in the HIV-infected aged 50–59 subgroup. Sacktor noted that the age-specific increase in amyloid deposition could suggest a “pre-clinical” stage of HIV-related neurocognitive disease, but there is as yet insufficient evidence for this, and further longitudinal imaging and cognitive testing of the age groups are warranted by this initial study (Sacktor 2018).

These structural studies support the notion of premature brain aging in the patient living with HIV infection. To better quantitate this, Cole et al. developed a “brain predicted age” from T1-weighted MRI scans of 2001 healthy individuals aged between 18 and 90 years, analyzing gray matter and white matter images together to quantify the normal whole brain (Cole et al. 2017). Using these normals, the investigators created a reference plot of brain-predicted age (years) versus chronological age (years), then went on to generate brain-predicted age difference (brain-PAD) scores by subtracting chronological age from the imaging-determined brain-predicted age. In 162 HIV-seropositive versus 105 “highly comparable” SN controls, the HIV-seropositive individuals with undetectable viral loads after at least a year of ART had higher brain-PAD scores, and therefore had “older” brains, by about 2.15 years. In addition, higher brain-PAD scores were associated with poorer cognitive performance. Interestingly, PAD scores did not interact with chronological age or duration of infection. The authors interpreted these results as favoring accentuated rather than accelerated structural aging in the HIV-infected brain.

Within the past two decades, proton MR spectroscopy (MRS) has been used to study neurometabolite levels in different brain regions of individuals with AIDS dementia complex (ADC) versus neuroasymptomatic HIV infection versus SN controls. Extensive data from both ART-naïve and primarily ART-treated subjects have indicated that advancing cognitive impairment is associated with significantly increasing glial metabolite MI/tCr (myoinositol-to-total creatine) or Cho/tCr (choline-to-total creatine) ratios in the basal ganglia and frontal white matter. This suggests glial activation and increased inflammation. With symptomatic neurocognitive impairment, neuronal metabolite NA/tCr (*N*-acetyl aspartate-to-total creatine) ratios are decreased, suggesting neuronal loss even at younger (< 40 years) age (Chang L 2012; Chang et al. 2004; Ernst and Chang 2004). HIV-infected subjects with symptomatic neurocognitive impairment also had lower NA/tCr ratios in the frontal white matter. Symptomatic neurocognitive impairment in HIV-infected subjects has correlated with the highest levels of MI/tCr and CHO/tCr in the basal ganglia across age groups (< versus ≥ 40 years), as compared to neuroasymptomatic HIV-infected subjects. And, in neuroasymptomatic subjects < 40 years old, HIV infection associated with higher MI/tCr and CHO/tCr ratios in basal ganglia compared to age-matched SN controls. The young neuroasymptomatic HIV-infected subjects’ ratios were like those of older (≥ 40 years) SN subjects. These patterns have suggested that there may be greater inflammatory response in younger HIV-infected subjects, but glial activation (with elevated MI in the frontal white matter) persists across the age span (Chang et al. 2004). MRS study of slightly older (median age 40–45 years) and ART-treated subjects showed MI/tCr increased with age in mildly cognitively impaired subjects but decreased with age in more severe brain disease such as ADC (Harezlak et al. 2011). This pattern may reflect an increase in microglia-driven inflammation in mildly impaired subjects, an accentuated aging effect, but premature microglial inflammatory senescence in advanced brain disease, e.g., accelerated aging (Holt et al. 2012). MRS study of glutamate found levels were lower even in younger HIV-infected subjects, similar to those of SN controls 10 years older (Ernst et al. 2010). Lower brain glutamate was associated with worsening cognitive deficits. Thus, while interpretation in some cases is complicated by the cross-sectional nature of the studies, brain metabolite data in HIV-infected subjects suggests features of both accentuated and accelerated aging effects leading to premature brain aging.

## Molecular markers associated with HIV and aging

Telomere length is a molecular chromosomal marker that correlates inversely with aging in normal populations. It has also been used to investigate aging in the HIV-infected population (Table 2). A study performed in South Africa that included 236 HIV-infected and 250 age-matched SN controls revealed that shorter telomeres were associated with HIV infection. In those infected individuals with undetectable viral loads, shorter telomeres were associated with lower CD4+ lymphocyte numbers (Pathai et al. 2013). Supporting studies by Zanet et al. (2014) have found a correlation between telomere length and HIV infection, age, peak viral loads higher than 100,000 copies/ml, and smoking, but not with ART. Similarly, no changes in rates of telomere shortening were found when HIV-infected individuals were switched from an ART regimen of darunavir/ritonavir supplemented with nonnucleoside reverse transcriptase inhibitor (NNRTI) to darunavir/ritonavir without NNRTI (Solomon et al. 2014). Telomere shortening occurred after seroconversion, but no further shortening was observed with prolonged infection (Leung et al. 2017), possibly suggesting accentuated aging as measured by telomere length.

**Table 2:** Studies linking HIV with molecular markers of aging

Publication	Study population	Measures	Findings
Pathai et al. (2013)	HIV+ *n* = 236 ^a^Age 39 years (35–46) 68% on ART SN *n* = 250 age 40 years (35–49)	Telomere length	Shorter telomeres associated with HIV infection and with lower CD4+ cell numbers in ART-treated individuals with undetectable viral loads
Zanet et al. (2014)	HIV+ *n* = 229 median age 40 years 92% on ART SN *n* = 166 median age 38	Telomere length	Shorter telomeres associated with infection, older age, active HCV infection. No correlation with ART
Solomon et al. (2014)	HIV+ n = 124 Treated with darunavir/ritonavir *n* = 63, age 63 years Treated with darunavir/ritonavir+ NNRTI *n* = 61, age 40 yrs	Telomere length	No difference in telomere length between patients on darunavir/ritonavir with and without NNRTI
Rickabaugh et al. (2015)	Grouped by age and HIV serostatus all ART naive all groups n = 12 HIV+ 20–24 years SN 20–24 years HIV+ 48–56 years SN 48–56 years HIV+ 27–35 years SN 27–35 years HIV+ 36–56 years SN 36–56 years	DNA methylation	HIV infection accelerated age-associated methylation by 13.7 to 14.7 years
Horvath and Levine (2015)	Brain and blood tissues from different sources	DNA methylation	HIV infection increased epigenetic methylation in brain and blood tissue Epigenetic age increased 7.4 years in brain Epigenetic age increased 5.2 years in blood
Study population details: Data set 1: Brain specimens HIV+ *n* = 71, age at death 45.9 years, 91.5% on ART SN *n* = 13, age NS Data set 2: Brain specimens HIV+ *n* = 8, age 44 years; SN *n* = 44, age NS Data set 3: Cerebellar samples HIV+ *n* = 8 (same as data set 2); SN *n* = 12, age NS Data set 4: Blood samples HIV+ *n* = 24, age 49 years, no ART information; SN *n* = 68, age 36 years Data set 5: PBMC samples HIV+ *n* = 23, age 45 years; SN *n* = 69, age 51 years Data set 6: PBMC samples HIV+ *n* = 109, age 52 years Data set 7: Whole blood SN *n* = 335, age 17–70 years Data set 8: Sorted leukocytes SN *n* = 4, age NS Data set 9: Sorted blood sets SN = 6, age 38 years Data set 10: T cells and monocytes SN *n* = 23, age NS Data set 11: Sorted blood cells SN *n* = 6, age 36–51 years			
Gross et al. (2016)	Whole blood samples HIV+ *n* = 137 age 45.7 years all on ART SN *n* = 44 age 48.2 years Validation panel whole blood samples from HIV+ *n* = 35 age 45.85 SN *n* = 25 age 41.72 years	DNA methylation	HIV+ had advanced aging by 4.9 years Epigenetic age independent of duration of infection
Levine et al. (2016)	HIV+ *n* = 58 With HAND *n* = 43 age at death 45.7 years 77% on ART Neurocognitively normal *n* = 15 age at death 46.5 years 87% on ART	Retrospective analysis of neurocognitive tests within 1 year of death DNA methylation from post-mortem brain	HAND associated with increased methylation age of 3.5 years No correlation with global neurocognitive functioning or HAND severity Age acceleration correlated with duration of HIV infection
Leung et al. (2017)	31 IV drug users who seroconverted to HIV+ age 35.8 years 22% on ART by last time point	Longitudinal blood samples -Telomere length -DNA methylation	Lower telomere length and changes in methylation patterns within 2 years after seroconversion Telomeres were not further shortened over subsequent 2 years

*NS* age not specified, *PBMC* peripheral blood mononuclear cells, *ART* antiretroviral treatment

^a^Age values are mean ages unless otherwise specified as median or age range

In addition to telomere length, changes in DNA methylation patterns (the “epigenetic clock”) have been associated with the aging process (Alisch et al. 2012; Bollati et al. 2009) and with medical disease. Two models of age-associated methylation changes have recently been applied to the study of epigenetic aging in HIV infection. Horvath and Levine (2015) compiled methylation data from the blood cells and brain tissue of HIV-infected and uninfected individuals, while Hannum et al. (2013) compiled data from the whole blood cells of uninfected individuals of various ages. Gross et al. applied a combined model that included the age-associated methylation changes compiled by Horvath and Levine (2015) and those compiled by Hannum et al. (2013), and proposed that HIV advances biological aging by 4.9 years and increases mortality by 19% (Gross et al. 2016). Gross et al. adjusted for the different composition of cell types in whole blood from HIV-infected versus normal individuals. Additionally, the HIV-infected population studied was otherwise healthy and on ART, thus avoiding the confounding contributions to aging of medical comorbidities and coinfections. Interestingly, predicted biological age, as measured by the epigenetic clock, was independent of the duration of infection, suggesting an accentuated rather than accelerated aging. Of all the 81,361 DNA methylation CpG sites characteristic of HIV infection, 4338 were also associated with aging, indicating, as expected, that many other processes associated with HIV infection are being modified by methylation. This biological age difference between HIV-infected and uninfected individuals was lower than that published by Rickabaugh and collaborators—close to 14 years (Rickabaugh et al. 2015). Differences between the two studies in the prediction of biological aging due to HIV infection may be due to the use of a different model to calculate epigenetic age. Gross et al. used the average of the above-named two models to calculate epigenetic aging (Horvath and Levine 2015; Hannum et al. 2013), while Rickabaugh et al. used only the Horvath and Levine model. This biological age difference may also be due to the fact that, in the Rickabaugh et al. study, samples from antiretroviral-naïve individuals were used. An increase in CD4+ lymphocyte numbers is an expected outcome of ART (Kaufmann et al., 2003), and lower CD4+/CD8+ ratios are associated with increased epigenetic aging (Gross et al. 2016). Thus, ART may result in younger age, as measured by the epigenetic clock, due to an increase in CD4+ lymphocyte numbers. In addition, in a 2016 study conducted by Levine et al. (2016), 58 HIV+ individuals were diagnosed with or without HAND within 1 year of their death. Post-mortem brain tissue analysis was used to calculate biological age based on DNA methylation patterns (Horvath and Levine 2015), and determined that individuals with HAND had an increased biological age by a mean of 3.5 years as compared to individuals without HAND. However, as the age of the patient increases, the differences in biological aging between the HAND and the asymptomatic group widened, and increased biological age was associated with length of infection, consistent with accelerated aging. Interestingly, this biological age acceleration did not depend on the severity of HAND symptoms. These results suggest that molecular markers of aging can be used to evaluate the progression of HIV infection. But further longitudinal studies are needed to understand whether some of these molecular markers may herald neurological changes, and thus can be used as markers for earlier therapeutic interventions.

## Apolipoprotein E and the aging HIV-infected population

Apolipoprotein E (ApoE) is a small secreted protein involved in cellular lipid and cholesterol transport (Mahley 1988; Mahley and Rall 2000). In the brain, neuronal processes such as synaptogenesis and neurite extension are dependent on a supply of cholesterol and phospholipids, which are transported by ApoE after production and secretion by astrocytes (Boyles et al. 1985; Qiu et al. 2004). Thus, ApoE is vital for neurons; it is involved in neuronal development, migration, and survival. Three isoforms of ApoE exist in the human population, ApoE2, ApoE3, and ApoE4; they differ by the presence or absence of arginine or cysteine residues at amino acid positions 112 and 158. These amino acid substitutions result in distinct structural and functional differences, including binding to lipoproteins and to the ApoE receptor (Hatters et al. 2006; Hauser et al. 2011; Mahley et al. 2009; Yamamoto et al. 2008). Associated with ApoE isoforms E2, E3, and E4 are distinct genetic alleles, ε2, ε3, and ε4, respectively. The most common phenotype is E3/E3, with a prevalence of 60–63%, and the least frequent is E2/E2, with a prevalence of only 1% of individuals tested. Approximately 28% of the population carries at least one ε4 allele (reviewed by Mahley 1988).

Apolipoprotein E4 has been characterized as the single most prominent human host risk factor for developing Alzheimer’s disease (Corder et al. 1993; Michaelson 2014). Studies carried out primarily to find predictors of Alzheimer’s disease in aging subjects provide information about the influence of ApoE4 on cognitive function even in the absence of overt dementia and suggest that ApoE4 is associated with lower or longitudinally declining performance in different cognitive domains. In a longitudinal study of 369 educationally and ethnically diverse older adults, Apoε4 carriers had lower baseline scores and faster decline for the cognitive measure Episodic Memory, and Apoε4 was also related to decline of the cognitive measure Executive Function (Mungas et al. 2010). In a cross-sectional study of 105 subjects ≥ 60 years and with normal mental status screening, there was a significant relationship between Apoε4 and worse performance on verbal memory testing using the Word List Memory Immediate Recall (Turana et al. 2015). In a longitudinal study of “relatively high functioning” men and women aged 70 to 79 years (MacArthur Successful Aging Study), initial modest cognitive decline in naming and spatial ability was noted in Apoε4 carriers (Bretsky et al. 2003); by 7 years, Apoε4 carriers were twice as likely to have global cognitive score declines compared to noncarriers (subjects with no Apoε4 allele). In general, the acquisition risk as well as the prevalence risk of mild cognitive impairment have been associated with the Apoε4 allele in aging cohorts (DeCarli et al. 2001; Lipnicki et al. 2017; Roberts et al. 2015). The Apoε effect on cognitive impairment risk may be diminished somewhat by enriched education, which associates with higher frontal and temporal lobe metabolism, independent of amyloid deposition (Arenaza-Urquijo et al. 2015). This implies that the clinical effect of ApoE4 can be delayed or offset by greater cognitive reserve. Interestingly, ApoE genotype also affects survival: ε2 allele carriers live longer than individuals that are homozygous for the ε3 allele, and these live longer than carriers of the ε4 allele (Blanche et al. 2001; Rea et al. 2001; Schachter et al. 1994).

The influence of ApoE phenotype or genotype on the pathogenesis of HAND in HIV infection is controversial; conflicting results have emerged from numerous studies over the past two decades (Table 3). Given its role in neuronal structure and function, any impact of ApoE may be the product of multiple confounding factors, such as age-related effects of HIV infection, antiretroviral therapy, and medical comorbidities. Added to that, the complexity of symptoms that can lead to a diagnosis of HAND may complicate the analysis of possible associations with ApoE.

**Table 3:** Studies relating apolipoprotein E (ApoE) to HIV and aging

Publication	Study population	Measures	Findings
Dunlop et al. (1997)	HIV+ autopsied *n* = 132 ^a^Median age = 37 years Zidovudine only ART	Neuropsychological testing Senile plaques Amyloid deposits ApoE genotype	No correlation between ApoE genotype and HIV dementia or encephalitis
Diaz-Arrastia et al. (2004)	HIV+ *n* = 270, who died of AIDS most on ART Age at death not reported	Microscopic pathological exams of spinal cords ApoE genotype	No correlation between HIV encephalitis and ApoE genotype
Valcour et al. (2004b)	HIV+ *n* = 182 Ages > 50 years *n* = 97 97% on ART Ages 20–39 years *n* = 85 69% on ART	Neuropsychological tests ApoE genotype	Independent risk of HAD associated with Apoε4 in older but not younger individuals
Burt et al. (2008)	HIV+ *n* = 1267 369 Apoε4 carriers All accessed ART SN *n* = 1132 Similar frequency of Apoε4 carriers All subjects: age range 18–70 years, 94% male	Progression to death (survival curves) ApoE genotype Clinical history	Faster progression in Apoε3 noncarriers compared to Apoε3 carriers Fastest progression in Apoε4 homozygotes Progression similar in Apoε4 heterozygotes and Apoε4 noncarriers ε4 allele did not increase the risk for dementia
Joska et al. (2010)	HIV+ *n* = 144 age 29.7 years, ART naive SN *n* = 50 age 25.3 years	Neurological tests ApoE genotype	No correlation between ApoE genotype across different categories of HAND
Andres et al. (2011)	HIV+ *n* = 48, on ART 18 (38%) with HAND ε4+ *n* = 15, age 48.7 years ε4− *n* = 33, Age = 45.8 years 3 subjects ε4/ε4 homozygous SN *n* = 39 ε4+ *n* = 11, age 47.2 years ε4− *n* = 28, age 47.0 years 0 subjects ε4/ε4 homozygous	CSF ApoE isoform levels Cognitive tests ApoE genotype	Overall CSF ApoE levels lower in HIV+ than SN CSF ApoE levels similar in SNε4+ compared to SNε4− CSF ApoE levels higher in HIV+ ε4+ compared to HIV+ ε4− but were similar to SN levels In ε4+: higher CSF ApoE levels trended to worse learning or memory performance in both HIV+ and SN, but especially in HIV+
Chang et al. (2011)	HIV+ *n* = 69 Approximately 80% on ART 29 (42%) with HAND ε4− < 50 years *n* = 31 ε4− ≥ 50 years *n* = 16 ε4+ <50 years *n* = 11 ε4+ ≥50 years *n* = 11 SN *n* = 70 ε4− < 50 years *n* = 32 ε4− ≥ 50 years *n* = 22 ε4+ < 50 years *n* = 9 ε4+ ≥ 50 years *n* = 7	MRI morphometry Neuropsychological testing ApoE genotype	Brain volume: -HIV+ < SN -HIV+ ε4+ had the smallest brain volumes; younger SN ε4+ had the largest (putamen, cerebral white matter) Cognitive performance: -HIV+ ε4+ < HIV+ ε4− or SN (memory, learning, attention) -SN ε4+ > SN ε4− (memory) Ruff Figural Fluency Test: < 50 years SNε4+ better than < 50 years SNε4− < 50 years HIV+ ε4+ worse than < 50 years HIV+ ε4− > 50 years: no significant ε4-related differences in SN or HIV+ groups
Jahanshad et al. (2012)	HIV+ *n* = 55 SN *n* = 30 Age matched (60–80 years of age) All on ART	MRI plus DTI tractography ApoE genotype	HIV+ ε4+ have higher risk of neurodegeneration (impaired connectivity), exceeding normal aging Impaired connectivity in HIV+ ε4+ exacerbated with longer duration of infection
Soontornniyomkij et al. (2012)	HIV+ *n* = 160 cases from 4 cohorts Ages 27–67 years, median 46 years 63% on ART before death Frozen brain tissue from 151 SN *n* = 22 Ages 24–90 years, median 50 years	APOE genotyping *n* = 151 Immunohistochemistry for Aβ plaques *n* = 105 Neuropsychological test HAND *n* = 78	Probability and abundance of Aβ plaques is higher in Apoε4 carriers Apoε4 and age ≥ 50 years independently predict Aβ plaques Aβ plaques associated with HAND only with HIV+ Apoε4 carriers
Morgan et al. (2013)	HIV+ *n* = 466 Apoε4+ *n* = 144, age 44.3 years 68% on ART Apoε4− *n* = 322, age 44 years 69.9% on ART	Neurological tests ApoE genotype	No correlation between Apo ε4 and HAND
Hoare et al. (2013)	HIV+ *n* = 43 ε4+ *n* = 24 (21 heterozygous and 3 homozygous) Age 29.54 years ε4− *n* = 19 Age 28.58 years All subjects ART naive	Neuropsychological tests White matter integrity by DTI ApoE genotype	ε4 allele associated with memory impairment and corpus callosum atrophy
Panos et al. (2013)	HIV+ *n* = 259 ε4+ > 50 years *n* = 18 ε4+ < 50 years *n* = 59 ε4− > 50 years *n* = 39 ε4− < 50 years *n* = 143 Approximately 84.5% on ART	Neuropsychological testing ApoE genotype	No impact of ε4 on neurocognition in overall cohort HAND diagnosis more prevalent in older HIV+ε4+ than age-matched HIV+ε4− Older HIV+ε4+ showed reduced executive functions, processing speed, independent of disease severity Younger HIV+ε4+ and HIV+ε4− had similar frequency of HAND diagnosis
Chang et al. (2014)	HIV+ *n* = 80 Age 47.3 years ε4+ *n* = 23, age 47.0 years 91% on ART ε4− *n* = 57, age 47.4 years 93% on ART SN *n* = 97 Age 44.7 years ε4+ *n* = 28, age 45.3 years ε4− *n* = 69, age 44.5 years	MRS cross-sectional study Neuropsychological testing	HIV+: elevated myoinositol (MI) in frontal WM across the age span SN ε4+: antagonistic pleiotropy for MI ε4+: poor attention and working memory regardless of HIV status HIV ε4+: lowest performance on most cognitive tests across age span
Becker et al. (2015)	The Multicenter AIDS Cohort Study (MACS)	Neuropsychological testing Longitudinal analysis Time to impairment Age, Time to death (Cox regression) ApoE genotype	Significant increase in the prevalence of ε4 among non-white subjects (primarily African-American) ApoE genotype not associated with time to cognitive impairment and cognitive outcomes or with time to death ApoE genotype not significantly interacting with HIV to increase risk of death
Study population details: Cohort 1 (enrolled 1984 to 1985 and 1987 and 1991) *n* = 3411 with ApoE genotype: HIV+ *n* = 1224, SN *n* = 2187 Mean ages all subjects: Apoε4− non-white 32.09 years, Apoε4+ non-white 32.14 years, Apoε4− white 33.79 years, Apoε4+ white 33.55 years No ART Cohort 2 (enrolled 2001 to 2003) *n* = 864 with ApoE genotype: HIV+ *n* = 465, SN *n* = 399 Mean ages all subjects: Apoε4− non-white 38.29 years, Apoε4+ non-white 39.02 years, Apo4ε- white 36.78 years, Apo4ε+ white 38.47 years 364 on ART (78%) Total all subjects = 4275; 2846 of 4275 had neuropsychological evaluations 2204 of 2846 were cognitively normal at first evaluation 1481 of 2204 had no potentially confounding conditions for neurocognitive impairment			
Wendelken et al. (2016)	HIV+ *n* = 87 Ages 60–84 years 76 with ApoE genotype	Neuropsychological testing Brain MRI and DTI ApoE genotype	At least 1 ε4 allele associated with poorer cognitive test scores (executive function), reduced brain white matter integrity and brain atrophy (corpus callosum, thalamus)

*ART* antiretroviral treatment, *Aβ* amyloid beta

^a^Age values are mean ages unless otherwise specified as median or age range

Based on neuropsychological assessments, no correlation was found between the presence of Apoε4 alleles and HAND or Apoε4 alleles and dementia in relatively large, well-characterized HIV-infected cohorts (Becker et al. 2015; Joska et al. 2010; Morgan et al. 2013). Even though Burt et al. found a correlation between Apoε4/ε4 genotype and faster disease progression to death, they did not find an increased risk for HAD in all Apoε4 carriers (Burt et al. 2008). However, an earlier prospective study of a HIV-infected cohort with mean absolute CD4+ cell counts of approximately 350/mm^3^, “sufficient to avoid opportunistic infection” (Corder et al. 1998), reported a statistically significant excess of cognitive impairment symptoms and peripheral neuropathy in individuals with the ApoE4 isoform, some of whom had been treated with zidovudine ART.

Other studies have reported that the ε4 allele is associated with cognitive impairment in the context of HIV infection. In a cross-sectional study of 43 treatment naïve, young (≤ 35 years) HIV-infected adults in South Africa, Hoare and collaborators (2013) found that immediate and delayed verbal recall, determined by the Hopkins Verbal Learning Test-revised (HVLT-R), was significantly impaired in the 24 subjects with at least one Apoε4 allele compared to 19 subjects with no Apoε4 allele. Additionally, brain imaging with DTI detected atrophy of the corpus callosum in the Apoε4 carriers compared to the noncarriers. The investigators further stratified the study cohort into three groups: Apoε4 homozygotes (*n* = 3), Apoε4 heterozygotes (*n* = 24), and noncarriers (*n* = 19). Kruskal-Wallis tests of HVLT-R performed among the three groups produced a significant result, suggesting that verbal learning performance differed significantly across the three groups. While direct pairwise comparisons were not reported, the mean values of HVLT-R performance were lowest in the Apoε4 homozygous group, implying an ε4 allele dose effect, though interpretation is limited by the small number of homozygous Apoε4 subjects. The homozygous Apoε4 subjects also showed worsened corpus callosal atrophy compared to other subjects. Of note, all these patients were infected with clade C HIV-1, rather than the clade B HIV-1 infection of other reported cohorts. Chang et al. (2011) studied 139 “relatively young” subjects (average age < 50 years) by morphometry of MRI images to evaluate brain volumes (see below) as well as neuropsychological testing and ApoE genotyping (Chang et al. 2011). Their cohort included 69 HIV-infected and 70 SN subjects divided into four subject groups: HIV-infected Apoε4 carriers, HIV-infected noncarriers, SN Apoε4 carriers, SN noncarriers. The HIV-infected and SN subject groups had similar ages and prevalence of the Apoε4 allele; 29 (42%) of HIV-infected subjects had HAND while 4 (6%) of SN controls had cognitive deficits “comparable to HAND.” Chang et al. did observe poorer cognitive performance (verbal fluency, learning, executive function, memory) in HIV-infected Apoε4 carriers compared to HIV-infected noncarriers and SN controls. In contrast, SN Apoε4 carriers scored significantly higher on verbal learning and memory testing compared to SN noncarriers. When subjects were divided into younger (< 50 years) versus older (≥ 50 years) subgroups, there was no significant Apoε4 effect in older HIV-infected or SN subjects, rather the Apoε4 effect was seen in younger subjects. In a study that estimated brain ApoE exposure as measured by ApoE levels in CSF, Andres et al. (2011) found that CSF ApoE levels were lower in HIV-infected versus healthy SN controls (mean age approximately 47–49 years). However, HIV-infected Apoε4 carriers had higher CSF ApoE levels, comparable to those of the SN controls, and the higher levels correlated with lower cognitive performance (HIV Dementia Scale and Global Cognitive Score). The investigators described this as a negative “dose-dependent” effect of CSF ApoE on cognition in HIV-infected Apoε4 carriers. Even SN Apoε4 carriers trended to an inverse relationship between CSF ApoE level and performance on tests related to learning, memory, and speed of processing. By contrast, in HIV-infected Apoε4 noncarriers, higher CSF ApoE levels trended to better performance on processing speed and executive functioning tests. But, there was no significant relationship between global cognitive scores and CSF ApoE level in either HIV-infected or SN without an Apoε4 allele. Thus, the effect of CSF ApoE levels in noncarriers was inconclusive.

The seemingly contradictory findings regarding the influence of the Apoε4 allele on cognitive performance in these HIV-infected cohorts may be due, at least in part, to an age dependence of the Apoε4 effect. Indeed, some studies have found an age differential of the Apoε4 effect when subjects older than 50–60 years were analyzed separately, while the “negative” studies examined aggregate cohorts less than 65 years of age (noted in Becker et al. 2015). In their study of the HAHC, Valcour et al. hypothesized that, since the association between Alzheimer’s disease and the Apoε4 allele was more pronounced in elderly patients, any association between the Apoε4 allele and neurocognitive decline may also manifest preferentially in older HIV-infected patients. In their cohort of 182 HIV-infected participants, 97 were older than 50 years. Only in this older group was there an independent increased risk (odds ratio) for HAD associated with at least one ε4 allele, even after controlling for diabetes status and age (Valcour et al. 2004a; Valcour et al. 2004b). Panos et al. (2013) studied 259 HIV-infected subjects with average age of 43 years in both ApoE4 carriers and noncarriers. While there was no impact of the Apoε4 allele on neurocognitive functioning in the overall cohort, the older (≥ 50 years) Apoε4 carriers showed a significantly disproportionate risk (odds ratio = 13.14) for diagnosis of HAND compared with age-matched Apoε4 noncarriers. In the cognitive domains of executive functions and information processing speed, older Apoε4 carriers showed reduced performance, independent of disease severity (CD4+ cell count and duration of HIV infection). The younger Apoε4 carriers and Apoε4 noncarriers had a similar frequency of HAND diagnoses (odds ratio = 0.80).

In studies using pathological outcome measures, ApoE genotype did not correlate with neuropathological findings of HIV encephalitis or vacuolar myelopathy in earlier studies of AIDS patients (Diaz-Arrastia et al. 2004; Dunlop et al. 1997). But a more recent clinicopathological study of prospective cohorts including 160 HIV-infected patients found that the Apoε4 allele and older age (≥ 50 years) were independent factors associating with cortical (mid-frontal gyrus) amyloid-beta (Aβ) plaques (Soontornniyomkij et al. 2012). Plaques were diffuse and rarely associated with phospho-Tau immunoreactivity, and thus more characteristic of aging, not symptomatic Alzheimer’s disease. The Apoε4 allele was associated with the abundance of cerebral Aβ plaques, which is consistent with neuropathology in cognitively normal aging (Morris et al. 2010). But when considering the clinical data for the HIV-infected patients, there was an association between cerebral Aβ deposition and manifestation of HAND in the Apoε4 carriers, but not the noncarriers. This result had parallels to a study of 408 “cognitively normal,” uninfected older adults (median age 79 years) in whom Aβ plaque load was measured by PET scan with [11C] Pittsburgh compound B (PiB) (Kantarci et al. 2012). The global cortical Aβ load was increased in Apoε4 carriers, and greater Aβ load was associated with worse cognitive performance on testing for memory, attention, language, and visual processing. This association was strongest in the Apoε4 carriers compared to other ApoE genotypes. Both studies suggest that Apoε4 associated with greater cerebral Aβ load which in turn associated with poorer cognitive performance on testing. But in the HIV-infected cohort studied by Soontornniyomkij et al. (2012), symptomatic cognitive performance deficit occurred at a younger age, with HAND detected at median age of 44 years. In the uninfected, “cognitively normal” older adults studied by Kantarci et al., the cognitive performance decline was measured at median age 79 years. Although the two studies use different measures of Aβ load, their comparative results imply that HIV infection can accelerate cognitive aging in individuals who carry the Apoε4 allele.

Neuroimaging studies have also been used to examine the influence of ApoE4 on brain structure and metabolism in aging HIV infection. In a sample of older HIV-infected subjects aged 60–84, the presence of at least one Apoε4 allele was associated with decreased executive functioning on cognitive testing, with reduced brain white matter integrity, and with brain atrophy most prominent in the posterior corpus callosum and thalamus, as measured by MRI with DTI imaging (Wendelken et al. 2016). This cross-sectional study did not include SN controls but compared Apoε4 carriers to noncarriers. In a study using combined MRI-based cortical parcellations plus DTI tractography, brain degeneration beyond that expected for normal aging was found in HIV-infected, Apoε4 carriers between 60 and 80 years of age, as compared to age-matched SN (Jahanshad et al. 2012). Brain degeneration was assessed by mapping white matter connections between cortical regions of the brain. The study revealed that older HIV-infected Apoε4 carriers have a higher risk of impaired connectivity, particularly involving the temporal and parietal regions of the brain. Interestingly, interruptions in brain networks were exacerbated with longer duration of infection, implying an accelerated effect of HIV and Apoε4 on biological aging. The study by Chang and collaborators that used morphometry of MRI images to evaluate brain volumes (Chang et al. 2011) showed that global cerebral volumes were larger in SN individuals and smaller in the HIV-infected (− 4.8%, *p* < 0.001). The largest brain volumes were found in younger (< 50 years) SN Apoε4 carriers, while the smallest were found in the HIV-infected Apoε4 carriers. Significant HIV status and Apoε4 effects on brain volume were evident in multiple brain regions: the right amygdala, right palladium, left thalamus, left cerebellar white matter, bilateral cerebral white matter, right cerebral cortex, and global cerebral volume. When the imaging data were stratified for age, all older Apoε4 carriers (HIV-infected and SN ≥ 50 years) had smaller brain volumes than noncarriers. However, younger (< 50 years) SN Apoε4 carriers had larger brain volumes, larger than both younger SN without the Apoε4 allele and the older SN Apoε4 carriers. The younger (< 50 years) HIV-infected Apoε4 carriers showed atrophy particularly in the putamen and right cerebral white matter, when compared to the younger HIV-infected noncarriers; but this relative brain volume loss was not seen when comparing older HIV-infected Apoε4 carriers versus noncarriers. All SN and the HIV-infected noncarriers showed age-related volume decline in putamen and thalamus, while HIV-infected Apoε4 carriers did not, possibly because these structures had already atrophied at a younger age in those subjects (Chang et al. 2011). Thus, the presence of the Apoε4 allele appeared to accelerate aging in these HIV-infected Apoε4 carriers with respect to volume decline in certain brain regions. As noted above, the group of HIV-infected Apoε4 carriers also showed worse cognitive performance, and this was regardless of HAND diagnosis. The SN Apoε4 controls showed better memory, more so in the younger subjects. The investigators interpreted these findings as a demonstration of antagonistic pleiotropy by the Apoε4 allele, with a beneficial effect on younger but not older SN individuals, reflecting a differential impact of the Apoε4 allele during different life stages (Tuminello and Han 2011). However, a negative impact of the Apoε4 allele is seen much earlier in the HIV-infected subjects, suggesting an accelerated aging effect. A subsequent study using MRS (Chang et al. 2014) showed antagonistic pleiotropic effects of the Apoε4 allele on the frontal lobe white matter myo-inositol, the marker for glial activation and neuroinflammation, in the SN subjects. Myo-inositol increased with age in all SN subjects, but with a lower initial value (at approximately age 25 years) and subsequent steeper slope between ages 25–70 years in the SN Apoε4 carriers compared to SN noncarriers. This suggests SN Apoε4 carriers have relatively less frontal lobe neuroinflammation at a young age, but “catch up” or even surpass the noncarriers at an older age, consistent with antagonistic pleiotropy. In HIV-infected subjects, frontal white matter myo-inositol was increased across the age span, regardless of ApoE genotype, suggesting that HIV infection attenuated the antagonistic pleiotropic effect of Apoε4 on neuroinflammation. As antagonistic pleiotropy by the Apoε4 allele can produce counterintuitive findings in SN Apoε4 carriers, it may be another factor underlying seemingly contradictory findings on the influence of the Apoε4 allele in studies of HIV-infected versus SN subjects, particularly if clinical studies do not have adequate younger and older age representation in both the HIV-infected and SN control groups.

## ApoE4 and HIV infection: potential mechanisms of interaction

Studies in rodent models or mammalian cell cultures have produced diverse molecular mechanisms that illustrate a differential effect of ApoE4 on neural cells and the brain, and the potential for interaction with HIV infection (Supplementary Table S1). Rodent models have used targeted replacement (TR) mice, which are transgenic animals generated by microinjecting the human ApoE gene (either Apoε2, ε3, or ε4) into single-celled embryos fertilized by APOE knockout males. These mice are then backbred with ApoE knockout mice to generate heterozygous mice for human ApoE gene but lacking the mouse ApoE. Continuous breeding of heterozygous mice is performed to generate homozygous human ApoE mice. ApoE expression is driven by a human ApoE promoter with a pattern that is similar to that of ApoE in humans (Xu et al. 1996). These mice, “humanized” for ApoE expression, reproduced differential ApoE4 effects on such processes as dendritic complexity (Dumanis et al. 2009), innate neuroimmune responses (Vitek et al. 2009), neuronal regeneration following innate immune inflammation (Maezawa et al. 2006c), or neuronal responses to traumatic brain injury (TBI) (Crawford et al. 2009; Sabo et al. 2000). Primary microglia cultures from TR ApoE mice stimulated with lipopolysaccharide (LPS) displayed an isoform-dependent innate immune activation that was highest with TR ApoE4 and dependent on p38MAPK signaling (Maezawa et al. 2006b), but the primary astrocytes from these mice showed lower levels of secreted inflammatory cytokines, attributed to differences in NF-κB signaling (Maezawa et al. 2006a). In vivo stimulation of innate immunity by intracerebroventricular LPS injection of TR ApoE mice resulted in loss of dendrite length in hippocampal neurons after 24 h, similar for all three ApoE isoforms. However, recovery of dendrite length over the subsequent 48 h was ApoE isoform dependent, and TR ApoE4 mice did not display any observable dendrite regeneration (Maezawa et al. 2006c). In a study of controlled impact TBI, rodents expressing the human ApoE4 died more frequently, while the ApoE4 survivors showed worse cognitive function and decreased neurite growth compared to TR mice expressing human ApoE3 protein or no human ApoE (Buttini et al. 1999; Sabo et al. 2000). After TBI, ApoE3 and ApoE4 mice showed differences in gene expression profiles from both cortex and hippocampus (Crawford et al. 2009), and the differences suggested a “loss of reparative function rather than a gain of negative function” in the presence of ApoE4. Recent studies using human neural cell cultures underscore the potential for repressive effects of ApoE4 on gene transcription. In a study using human neuroblastoma and human glioblastoma cells, transfection with ApoE3 and ApoE4 expression vectors demonstrated that ApoE4 interacted with certain specific gene promoters, reducing transcription, and reducing the mRNA levels for these genes (Theendakara et al. 2016). This implied a capacity forApoE4 to function as a transcriptional repressor. To identify specific effects of ApoE isoforms on neurons in the context of HIV infection, human neuronal lineage progenitor cells with the Apoε3/ε3 genotype were differentiated in culture with added HIV or control supernatants and individual recombinant ApoE isoform proteins rApoE3 or rApoE4. While addition of rApoE3 resulted in discrete changes in gene expression, addition of rApoE4 resulted in the downregulation of 85 genes, half of which involved processes associated with neurogenesis, an effect that was magnified in the presence of HIV (Geffin et al. 2017). Both of these studies used a strategy of altering the ApoE phenotype in human neural cells in culture, producing a common outcome of depressed differential gene expression associated with the ApoE4 phenotype compared to the ApoE3 phenotype.

ApoE4 may also interact directly with HIV proteins or infected cells to increase the net neurotoxic effects of neural infection. In an in vitro study using neuronal cultures derived from 54 different human fetal brain specimens, neurotoxicity, measured by decreased mitochondrial potential, was observed in cells exposed to gp120 plus Tat or Tat plus morphine. Cultures with the Apoε3/ε4 or Apoε4/ε4 genotypes were significantly more susceptible to toxic effects, with a threefold increase in neurotoxicity detected in the Apoε4/ε4 cells exposed to gp120 plus Tat (Turchan-Cholewo et al. 2006). Indeed, tat protein, binds to the neuronal lipoprotein receptor LRP1, which can inhibit uptake and clearance of ApoE4, effectively prolonging its circulation in the neuron’s microenvironment. Viral gp120 may affect ApoE binding at the cell surface, as suggested by data showing that added rApoE4 but not rApoE3-enhanced HIV-target lymphoid cell fusion and increased cell susceptibility to HIV infection (Burt et al. 2008). ApoE4 may also augment the impact of HIV infection on Aβ beta production (Pulliam 2009) and further accelerate the impact of aging on the HIV-Aβ dynamic.

## Conclusions

Now, well into the third decade of the use of increasingly effective ART, HIV infection is not a death sentence when adequately diagnosed and treated. HIV-infected individuals will live longer, often into the seventh or eighth decades of life. HIV infection has become a chronic medical and neurological disorder. As such, it is now a disorder that must be included in the differential diagnosis during clinical assessment of NCI in the older patient. While the severe dementia of HAD is far less prevalent, varying degrees of NCI are more prevalent in the HIV-infected population. Neuropsychological symptoms of NCI are variable among and within individuals (Hines et al. 2016), but an extensive body of neuropsychological evidence suggests that the HIV-infected person is cognitively older than a seronegative person of similar age and medical health. With effective ART, the clinical course of HAND likely reflects the compound effects of age-related decline in neurocognitive reserve (Chang et al. 2013), genetic factors, medical comorbidities, and treatment efficacy all superimposed on the underlying HIV infection. Paraclinical evidence, e.g., neuropathology or brain imaging studies, indicates that premature aging of the brain may be present even before neurocognitive symptoms. Age and HIV infection together impact brain volume loss, deposition of Aβ, and metabolic markers reflecting glial or neuronal function. Premature brain aging is likely a combination of accentuated and accelerated aging effects. Accentuated aging would be evident throughout the time course of infection, such as the higher brain-PAD scores reflecting structurally older brains (Cole et al. 2017), or the older biological age, independent of duration of infection, measured by epigenetic DNA methylation (Gross et al. 2016). Accelerated aging would be increasingly evident with age, such as the time-related changes manifest in DTI patterns of the corpus callosum genu (Chang et al. 2008).

The experimental evidence supporting the concept of premature brain aging in HIV infection has been confounded by the relatively young age of study cohorts, particularly in the pre-ART and early ART eras. Moreover, SN controls were not consistently matched for medical comorbidities or health risk factors which are common to aging. As studies advanced into the second and third decades of the ART era, improved survival produced HIV-infected study populations that were better stratified into younger and older subgroups, roughly ≤ or ≥ 50 years, with corresponding age-matched SN groups that are also controlled for medical comorbidities. The introduction of much older study subjects, roughly ≥ 65 years, has clarified the recognition that cognitive performance in younger HIV-infected individuals is similar to that of much older SN individuals, a finding that suggests accelerated aging. Neuropsychological testing and neuroimaging modalities have proved sensitive enough to detect abnormalities that are “pre-clinical” or “paraclinical,” i.e., present in HIV-infected individuals who are neuroasymptomatic. Overall, the evidence from more than three decades of studies support the concept of HIV infection as increasing the risk for premature brain aging, manifested by cognitive impairment or brain volume loss. But HIV infection does not cause either pre-clinical or symptomatic Alzheimer’s disease in the aging patient, and that distinction must be considered in clinical assessments of patients who present with cognitive complaints.

ApoE4 is emerging as a factor influencing cognitive function in normal aging, showing differential effects across the lifespan, or “antagonistic pleiotropy.” These effects may be beneficial in younger individuals but pathological in older individuals. Neuroimaging studies indicate that brain structural and metabolic parameters decline at relatively younger ages in HIV-infected Apoε4 carriers. To this point, Chang and collaborators have presented compelling evidence that suggests that HIV infection negates the potentially beneficial effects of antagonistic pleiotropy on cognition, brain volume, or frontal white matter inflammation in younger Apoε4 carriers (Chang et al. 2011; Chang et al. 2014). But overall, neuropsychological studies of HIV-infected cohorts have produced conflicting results regarding the impact of ApoE4 or the Apoε4 allele on the course of cognition. Neuropsychological performance in HIV-infected subjects is a very complex outcome measure that is confounded by the individual’s age, medical comorbidities, genetic influences, and within-person variability (Hines et al. 2016), as well as the intensity of infection itself and treatment side effects. This makes neuropsychology-based studies difficult to design, conduct, analyze, and compare. The age dependence of ApoE4 effects, including antagonistic pleiotropy, likely underlies, at least to some extent, the conflicting neuropsychology study results. Only relatively recently have HIV-infected individuals been surviving into the sixth and seventh decades of life, thus enabling better comparisons with normal aging. In normal aging, the negative cognitive impact of ApoE4 increases with age; the same appears to be true in aging during HIV infection, though the negative impact may be evident a decade or more earlier.

Do ApoE4 and HIV infection interact directly to potentiate premature brain aging? Data provided by neuropsychology testing and neuroimaging studies are not conclusive; data show trends for interaction but do not achieve statistical significance. There is non-clinical data to model potential mechanisms of interaction between ApoE4 and HIV proteins or virions at the cellular level. Human cell culture experiments have demonstrated effects of ApoE4 on neuronal gene expression profiles after HIV exposure (Geffin et al. 2017), and the ApoE4 phenotype may potentiate a “loss of reparative function” (Crawford et al. 2009) or diminished neurogenesis at the gene expression level. In particular, ApoE4 may be capable of direct transcriptional repression for some genes (Theendakara et al. 2016). If ApoE4 impairs expression of genes related to neural repair or neuronal complexity, then its effects on the brain would be compounded by HIV infection and aging, leading ultimately to diminished cognitive reserve.

Together, the clinical and non-clinical data of the current ART era do provide sufficient basis to implicate Apoε4 carrier status as an additional longitudinal risk factor for premature brain aging in HIV infection. If the impact of the Apoε4 allele on brain aging and cognitive reserve can be better clarified as to its predictive value, then ApoE genotype may inform cognitive risk assessment and treatment planning for the aging HIV-infected patient. Thus, as the aging HIV-infected population grows, so does the need for further longitudinal studies of these patients, studies which include extended age ranges, both clinical and paraclinical outcome measures, and ApoE genotyping and stratification. Given the low frequency of the Apoε4 allele in the general population, such study designs present statistical challenges that would probably necessitate multicenter involvement. But ultimately, such studies can extend our understanding of cognitive reserve not only in aging HIV-infected but also in normal controls, and thus lead to better health care for the aging population.

## Electronic supplementary material

ESM 1 (DOC 85 kb)

## References

[CR1] Alisch RS, Barwick BG, Chopra P, Myrick LK, Satten GA, Conneely KN, Warren ST (2012). Age-associated DNA methylation in pediatric populations. Genome Res.

[CR2] Ances BM, Ortega M, Vaida F, Heaps J, Paul R (2012). Independent effects of HIV, aging, and HAART on brain volumetric measures. J Acquir Immune Defic Syndr.

[CR3] Andres MA, Feger U, Nath A, Munsaka S, Jiang CS, Chang L (2011). APOE epsilon 4 allele and CSF APOE on cognition in HIV-infected subjects. J NeuroImmune Pharmacol.

[CR4] Antiretroviral Therapy Cohort Collaboration (2017). Survival of HIV-positive patients starting antiretroviral therapy between 1996 and 2013: a collaborative analysis of cohort studies. Lancet HIV.

[CR5] Appay V, Sauce D (2017). Assessing immune aging in HIV-infected patients. Virulence.

[CR6] Arenaza-Urquijo EM, Gonneaud J, Fouquet M, Perrotin A, Mezenge F, Landeau B, Egret S, De la Sayette V, Desgranges B, Chetelat G (2015). Interaction between years of education and APOE epsilon4 status on frontal and temporal metabolism. Neurology.

[CR7] Avci G, Loft S, Sheppard DP, Woods SP (2016). The effects of HIV disease and older age on laboratory-based, naturalistic, and self-perceived symptoms of prospective memory: does retrieval cue type and delay interval matter?. Neuropsychol Dev Cogn B Aging Neuropsychol Cogn.

[CR8] Bachis A, Aden SA, Nosheny RL, Andrews PM, Mocchetti I (2006). Axonal transport of human immunodeficiency virus type 1 envelope protein glycoprotein 120 is found in association with neuronal apoptosis. J Neurosci.

[CR9] Bachis A, Avdoshina V, Zecca L, Parsadanian M, Mocchetti I (2012). Human immunodeficiency virus type 1 alters brain-derived neurotrophic factor processing in neurons. J Neurosci.

[CR10] Becker JT, Lopez OL, Dew MA, Aizenstein HJ (2004). Prevalence of cognitive disorders differs as a function of age in HIV virus infection. AIDS.

[CR11] Becker JT, Martinson JJ, Penugonda S, Kingsley L, Molsberry S, Reynolds S, Aronow A, Goodkin K, Levine A, Martin E, Miller EN, Munro CA, Ragin A, Sacktor N (2015). No association between Apoepsilon4 alleles, HIV infection, age, neuropsychological outcome, or death. J Neurovirol.

[CR12] Bhatia R, Ryscavage P, Taiwo B (2012). Accelerated aging and human immunodeficiency virus infection: emerging challenges of growing older in the era of successful antiretroviral therapy. J Neurovirol.

[CR13] Blanche H, Cabanne L, Sahbatou M, Thomas G (2001). A study of French centenarians: are ACE and APOE associated with longevity?. C R Acad Sci III.

[CR14] Bollati V, Schwartz J, Wright R, Litonjua A, Tarantini L, Suh H, Sparrow D, Vokonas P, Baccarelli A (2009). Decline in genomic DNA methylation through aging in a cohort of elderly subjects. Mech Ageing Dev.

[CR15] Boyles JK, Pitas RE, Wilson E, Mahley RW, Taylor JM (1985). Apolipoprotein E associated with astrocytic glia of the central nervous system and with nonmyelinating glia of the peripheral nervous system. J Clin Invest.

[CR16] Bretsky P, Guralnik JM, Launer L, Albert M, Seeman TE (2003). The role of APOE-epsilon4 in longitudinal cognitive decline: MacArthur Studies of Successful Aging. Neurology.

[CR17] Burt TD, Agan BK, Marconi VC, He W, Kulkarni H, Mold JE, Cavrois M, Huang Y, Mahley RW, Dolan MJ, McCune JM, Ahuja SK (2008). Apolipoprotein (apo) E4 enhances HIV-1 cell entry in vitro, and the APOE epsilon4/epsilon4 genotype accelerates HIV disease progression. Proc Natl Acad Sci U S A.

[CR18] Buscemi L, Ramonet D, Geiger JD (2007). Human immunodeficiency virus type-1 protein Tat induces tumor necrosis factor-alpha-mediated neurotoxicity. Neurobiol Dis.

[CR19] Buttini M, Orth M, Bellosta S, Akeefe H, Pitas RE, Wyss-Coray T, Mucke L, Mahley RW (1999). Expression of human apolipoprotein E3 or E4 in the brains of Apoe−/− mice: isoform-specific effects on neurodegeneration. J Neurosci.

[CR20] Chang L (2018). Themed discussion: aging brain and HIV: before and after. Conference on retroviruses and opportunistic infections; March 6, 2018.

[CR21] Chang L, Lee PL, Yiannoutsos CT, Ernst T, Marra CM, Richards T, Kolson D, Schifitto G, Jarvik JG, Miller EN, Lenkinski R, Gonzalez G, Navia BA (2004). A multicenter in vivo proton-MRS study of HIV-associated dementia and its relationship to age. Neuroimage.

[CR22] Chang L, Wong V, Nakama H, Watters M, Ramones D, Miller EN, Cloak C, Ernst T (2008). Greater than age-related changes in brain diffusion of HIV patients after 1 year. J NeuroImmune Pharmacol.

[CR23] Chang L, Andres M, Sadino J, Jiang CS, Nakama H, Miller E, Ernst T (2011). Impact of apolipoprotein E epsilon4 and HIV on cognition and brain atrophy: antagonistic pleiotropy and premature brain aging. Neuroimage.

[CR24] Chang L, Feger U, Ernst T, Gendelman HE, Grant I, Everall IP, Fox HS, Gelbard HA, Lipton SA, Swindells S (2012). Bioimaging. The neurology of AIDS.

[CR25] Chang L, Holt JL, Yakupov R, Jiang CS, Ernst T (2013). Lower cognitive reserve in the aging human immunodeficiency virus-infected brain. Neurobiol Aging.

[CR26] Chang L, Jiang C, Cunningham E, Buchthal S, Douet V, Andres M, Ernst T (2014). Effects of APOE epsilon4, age, and HIV on glial metabolites and cognitive deficits. Neurology.

[CR27] Cole JH, Underwood J, Caan MW, De Francesco D, van Zoest RA, Leech R, Wit FW, Portegies P, Geurtsen GJ, Schmand BA, Schim van der Loeff MF, Franceschi C, Sabin CA, Majoie CB, Winston A, Reiss P, Sharp DJ (2017). Increased brain-predicted aging in treated HIV disease. Neurology.

[CR28] Corder EH, Saunders AM, Strittmatter WJ, Schmechel DE, Gaskell PC, Small GW, Roses AD, Haines JL, Pericak-Vance MA (1993). Gene dose of apolipoprotein E type 4 allele and the risk of Alzheimer’s disease in late onset families. Science.

[CR29] Corder EH, Robertson K, Lannfelt L, Bogdanovic N, Eggertsen G, Wilkins J, Hall C (1998). HIV-infected subjects with the E4 allele for APOE have excess dementia and peripheral neuropathy. Nat Med.

[CR30] Crawford F, Wood M, Ferguson S, Mathura V, Gupta P, Humphrey J, Mouzon B, Laporte V, Margenthaler E, O'Steen B, Hayes R, Roses A, Mullan M (2009). Apolipoprotein E-genotype dependent hippocampal and cortical responses to traumatic brain injury. Neuroscience.

[CR31] Currier JS, Lundgren JD, Carr A, Klein D, Sabin CA, Sax PE, Schouten JT, Smieja M (2008). Epidemiological evidence for cardiovascular disease in HIV-infected patients and relationship to highly active antiretroviral therapy. Circulation.

[CR32] Davis LE, Hjelle BL, Miller VE, Palmer DL, Llewellyn AL, Merlin TL, Young SA, Mills RG, Wachsman W, Wiley CA (1992). Early viral brain invasion in iatrogenic human immunodeficiency virus infection. Neurology.

[CR33] DeCarli C, Miller BL, Swan GE, Reed T, Wolf PA, Carmelli D (2001). Cerebrovascular and brain morphologic correlates of mild cognitive impairment in the National Heart, Lung, and Blood Institute Twin Study. Arch Neurol.

[CR34] Diaz-Arrastia R, Gong Y, Kelly CJ, Gelman BB (2004). Host genetic polymorphisms in human immunodeficiency virus-related neurologic disease. J Neurovirol.

[CR35] Dubrow R, Silverberg MJ, Park LS, Crothers K, Justice AC (2012). HIV infection, aging, and immune function: implications for cancer risk and prevention. Curr Opin Oncol.

[CR36] Dumanis S. B., Tesoriero J. A., Babus L. W., Nguyen M. T., Trotter J. H., Ladu M. J., Weeber E. J., Turner R. S., Xu B., Rebeck G. W., Hoe H.-S. (2009). ApoE4 Decreases Spine Density and Dendritic Complexity in Cortical Neurons In Vivo. Journal of Neuroscience.

[CR37] Dunlop O, Goplen AK, Liestol K, Myrvang B, Rootwelt H, Christophersen B, Kvittingen EA, Maehlen J (1997). HIV dementia and apolipoprotein E. Acta Neurol Scand.

[CR38] Ernst T, Chang L (2004). Effect of aging on brain metabolism in antiretroviral-naive HIV patients. AIDS.

[CR39] Ernst T, Jiang CS, Nakama H, Buchthal S, Chang L (2010). Lower brain glutamate is associated with cognitive deficits in HIV patients: a new mechanism for HIV-associated neurocognitive disorder. J Magn Reson Imaging.

[CR40] Faissner S, Ambrosius B, Schanzmann K, Grewe B, Potthoff A, Munch J, Sure U, Gramberg T, Wittmann S, Brockmeyer N, Uberla K, Gold R, Grunwald T, Chan A (2014). Cytoplasmic HIV-RNA in monocytes determines microglial activation and neuronal cell death in HIV-associated neurodegeneration. Exp Neurol.

[CR41] Fan Y, He JJ (2016). HIV-1 Tat induces unfolded protein response and endoplasmic reticulum stress in astrocytes and causes neurotoxicity through glial fibrillary acidic protein (GFAP) activation and aggregation. J Biol Chem.

[CR42] Fields JA, Dumaop W, Crews L, Adame A, Spencer B, Metcalf J, He J, Rockenstein E, Masliah E (2015). Mechanisms of HIV-1 Tat neurotoxicity via CDK5 translocation and hyper-activation: role in HIV-associated neurocognitive disorders. Curr HIV Res.

[CR43] Freiberg MS, Chang CC, Kuller LH, Skanderson M, Lowy E, Kraemer KL, Butt AA, Bidwell Goetz M, Leaf D, Oursler KA, Rimland D, Rodriguez Barradas M, Brown S, Gibert C, McGinnis K, Crothers K, Sico J, Crane H, Warner A, Gottlieb S, Gottdiener J, Tracy RP, Budoff M, Watson C, Armah KA, Doebler D, Bryant K, Justice AC (2013). HIV infection and the risk of acute myocardial infarction. JAMA Intern Med.

[CR44] Geffin R, Martinez R, de Las Pozas A, Issac B, McCarthy M (2017). Apolipoprotein E4 suppresses neuronal-specific gene expression in maturing neuronal progenitor cells exposed to HIV. J NeuroImmune Pharmacol.

[CR45] Gross AM, Jaeger PA, Kreisberg JF, Licon K, Jepsen KL, Khosroheidari M, Morsey BM, Swindells S, Shen H, Ng CT, Flagg K, Chen D, Zhang K, Fox HS, Ideker T (2016). Methylome-wide analysis of chronic HIV infection reveals five-year increase in biological age and epigenetic targeting of HLA. Mol Cell.

[CR46] Grulich AE, Jin F, Poynten IM, Vajdic CM (2011). HIV, cancer, and aging. Sex Health.

[CR47] Guaraldi G, Orlando G, Zona S, Menozzi M, Carli F, Garlassi E, Berti A, Rossi E, Roverato A, Palella F (2011). Premature age-related comorbidities among HIV-infected persons compared with the general population. Clin Infect Dis.

[CR48] Hannum G, Guinney J, Zhao L, Zhang L, Hughes G, Sadda S, Klotzle B, Bibikova M, Fan JB, Gao Y, Deconde R, Chen M, Rajapakse I, Friend S, Ideker T, Zhang K (2013). Genome-wide methylation profiles reveal quantitative views of human aging rates. Mol Cell.

[CR49] Harezlak J, Buchthal S, Taylor M, Schifitto G, Zhong J, Daar E, Alger J, Singer E, Campbell T, Yiannoutsos C, Cohen R, Navia B (2011). Persistence of HIV-associated cognitive impairment, inflammation, and neuronal injury in era of highly active antiretroviral treatment. AIDS.

[CR50] Hatters DM, Peters-Libeu CA, Weisgraber KH (2006). Apolipoprotein E structure: insights into function. Trends Biochem Sci.

[CR51] Hauser PS, Narayanaswami V, Ryan RO (2011). Apolipoprotein E: from lipid transport to neurobiology. Prog Lipid Res.

[CR52] Heaton RK, Franklin DR, Ellis RJ, McCutchan JA, Letendre SL, Leblanc S, Corkran SH, Duarte NA, Clifford DB, Woods SP, Collier AC, Marra CM, Morgello S, Mindt MR, Taylor MJ, Marcotte TD, Atkinson JH, Wolfson T, Gelman BB, McArthur JC, Simpson DM, Abramson I, Gamst A, Fennema-Notestine C, Jernigan TL, Wong J, Grant I (2011). HIV-associated neurocognitive disorders before and during the era of combination antiretroviral therapy: differences in rates, nature, and predictors. J Neurovirol.

[CR53] High Kevin P., Brennan-Ing Mark, Clifford David B., Cohen Mardge H., Currier Judith, Deeks Steven G., Deren Sherry, Effros Rita B., Gebo Kelly, Goronzy Jörg J., Justice Amy C., Landay Alan, Levin Jules, Miotti Paolo G., Munk Robert J., Nass Heidi, Rinaldo Charles R., Shlipak Michael G., Tracy Russell, Valcour Victor, Vance David E., Walston Jeremy D., Volberding Paul (2012). HIV and Aging. JAIDS Journal of Acquired Immune Deficiency Syndromes.

[CR54] Hines LJ, Miller EN, Hinkin CH, Alger JR, Barker P, Goodkin K, Martin EM, Maruca V, Ragin A, Sacktor N, Sanders J, Selnes O, Becker JT (2016). Cortical brain atrophy and intra-individual variability in neuropsychological test performance in HIV disease. Brain Imaging Behav.

[CR55] Hoare J, Westgarth-Taylor J, Fouche JP, Combrinck M, Spottiswoode B, Stein DJ, Joska JA (2013). Relationship between apolipoprotein E4 genotype and white matter integrity in HIV-positive young adults in South Africa. Eur Arch Psychiatry Clin Neurosci.

[CR56] Holt JL, Kraft-Terry SD, Chang L (2012). Neuroimaging studies of the aging HIV-1-infected brain. J Neurovirol.

[CR57] Horvath S, Levine AJ (2015). HIV-1 infection accelerates age according to the epigenetic clock. J Infect Dis.

[CR58] Jahanshad N, Valcour VG, Nir TM, Kohannim O, Busovaca E, Nicolas K, Thompson PM (2012). Disrupted brain networks in the aging HIV+ population. Brain Connect.

[CR59] Joshi MK, Liu HH (2000). Acute rhabdomyolysis and renal failure in HIV-infected patients: risk factors, presentation, and pathophysiology. AIDS Patient Care STDS.

[CR60] Joshi D, O'Grady J, Dieterich D, Gazzard B, Agarwal K (2011). Increasing burden of liver disease in patients with HIV infection. Lancet.

[CR61] Joska JA, Combrinck M, Valcour VG, Hoare J, Leisegang F, Mahne AC, Myer L, Stein DJ (2010). Association between apolipoprotein E4 genotype and human immunodeficiency virus-associated dementia in younger adults starting antiretroviral therapy in South Africa. J Neurovirol.

[CR62] Kantarci K, Lowe V, Przybelski SA, Weigand SD, Senjem ML, Ivnik RJ, Preboske GM, Roberts R, Geda YE, Boeve BF, Knopman DS, Petersen RC, Jack CR (2012). APOE modifies the association between Abeta load and cognition in cognitively normal older adults. Neurology.

[CR63] Kaufmann GR, Perrin L, Pantaleo G, Opravil M, Furrer H, Telenti A, Hirschel B, Ledergerber B, Vernazza P, Bernasconi E, Rickenbach M, Egger M, Battegay M (2003). CD4 T-lymphocyte recovery in individuals with advanced HIV-1 infection receiving potent antiretroviral therapy for 4 years: the Swiss HIV Cohort Study. Arch Intern Med.

[CR64] Kaul M, Zheng J, Okamoto S, Gendelman HE, Lipton SA (2005). HIV-1 infection and AIDS: consequences for the central nervous system. Cell Death Differ.

[CR65] Kovari H, Sabin CA, Ledergerber B, Ryom L, Worm SW, Smith C, Phillips A, Reiss P, Fontas E, Petoumenos K, De Wit S, Morlat P, Lundgren JD, Weber R (2013). Antiretroviral drug-related liver mortality among HIV-positive persons in the absence of hepatitis B or C virus coinfection: the data collection on adverse events of anti-HIV drugs study. Clin Infect Dis.

[CR66] Leung JM, Fishbane N, Jones M, Morin A, Xu S, Liu JC, MacIsaac J, Milloy MJ, Hayashi K, Montaner J, Horvath S, Kobor M, Sin DD, Harrigan PR, Man SF (2017). Longitudinal study of surrogate aging measures during human immunodeficiency virus seroconversion. Aging (Albany NY).

[CR67] Levine AJ, Quach A, Moore DJ, Achim CL, Soontornniyomkij V, Masliah E, Singer EJ, Gelman B, Nemanim N, Horvath S (2016). Accelerated epigenetic aging in brain is associated with pre-mortem HIV-associated neurocognitive disorders. J Neurovirol.

[CR68] Lipnicki DM, Crawford J, Kochan NA, Trollor JN, Draper B, Reppermund S, Maston K, Mather KA, Brodaty H, Sachdev PS (2017). Risk factors for mild cognitive impairment, dementia and mortality: the Sydney Memory and Ageing Study. J Am Med Dir Assoc.

[CR69] Liu Y, Liu H, Kim BO, Gattone VH, Li J, Nath A, Blum J, He JJ (2004). CD4-independent infection of astrocytes by human immunodeficiency virus type 1: requirement for the human mannose receptor. J Virol.

[CR70] Maezawa I, Maeda N, Montine TJ, Montine KS (2006). Apolipoprotein E-specific innate immune response in astrocytes from targeted replacement mice. J Neuroinflammation.

[CR71] Maezawa I, Nivison M, Montine KS, Maeda N, Montine TJ (2006). Neurotoxicity from innate immune response is greatest with targeted replacement of E4 allele of apolipoprotein E gene and is mediated by microglial p38MAPK. FASEB J.

[CR72] Maezawa I, Zaja-Milatovic S, Milatovic D, Stephen C, Sokal I, Maeda N, Montine TJ, Montine KS (2006). Apolipoprotein E isoform-dependent dendritic recovery of hippocampal neurons following activation of innate immunity. J Neuroinflammation.

[CR73] Mahley RW (1988). Apolipoprotein E: cholesterol transport protein with expanding role in cell biology. Science.

[CR74] Mahley RW, Rall SC (2000). Apolipoprotein E: far more than a lipid transport protein. Annu Rev Genomics Hum Genet.

[CR75] Mahley RW, Weisgraber KH, Huang Y (2009). Apolipoprotein E: structure determines function, from atherosclerosis to Alzheimer’s disease to AIDS. J Lipid Res.

[CR76] Marquine MJ, Umlauf A, Rooney AS, Fazeli PL, Gouaux BD, Paul Woods S, Letendre SL, Ellis RJ, Grant I, Moore DJ (2014). The veterans aging cohort study index is associated with concurrent risk for neurocognitive impairment. J Acquir Immune Defic Syndr.

[CR77] Marquine MJ, Montoya JL, Umlauf A, Fazeli PL, Gouaux B, Heaton RK, Ellis RJ, Letendre SL, Grant I, Moore DJ (2016). The Veterans Aging Cohort Study (VACS) index and neurocognitive change: a longitudinal study. Clin Infect Dis.

[CR78] McArthur JC, Brew BJ (2010). HIV-associated neurocognitive disorders: is there a hidden epidemic?. AIDS.

[CR79] McArthur JC, Steiner J, Sacktor N, Nath A (2010). Human immunodeficiency virus-associated neurocognitive disorders: mind the gap. Ann Neurol.

[CR80] Michaelson DM (2014). APOE epsilon4: the most prevalent yet understudied risk factor for Alzheimer’s disease. Alzheimers Dement.

[CR81] Mocchetti I, Bachis A, Avdoshina V (2012). Neurotoxicity of human immunodeficiency virus-1: viral proteins and axonal transport. Neurotox Res.

[CR82] Morgan EE, Woods SP, Letendre SL, Franklin DR, Bloss C, Goate A, Heaton RK, Collier AC, Marra CM, Gelman BB, McArthur JC, Morgello S, Simpson DM, McCutchan JA, Ellis RJ, Abramson I, Gamst A, Fennema-Notestine C, Smith DM, Grant I, Vaida F, Clifford DB (2013). Apolipoprotein E4 genotype does not increase risk of HIV-associated neurocognitive disorders. J Neurovirol.

[CR83] Morris JC, Roe CM, Xiong C, Fagan AM, Goate AM, Holtzman DM, Mintun MA (2010). APOE predicts amyloid-beta but not tau Alzheimer pathology in cognitively normal aging. Ann Neurol.

[CR84] Mungas D, Beckett L, Harvey D, Farias ST, Reed B, Carmichael O, Olichney J, Miller J, DeCarli C (2010). Heterogeneity of cognitive trajectories in diverse older persons. Psychol Aging.

[CR85] Nosheny Rachel L., Bachis Alessia, Aden Sadia A., De Bernardi Maria A., Mocchetti Italo (2006). Intrastriatal administration of human immunodeficiency virus-1 glycoprotein 120 reduces glial cell-line derived neurotrophic factor levels and causes apoptosis in the substantia nigra. Journal of Neurobiology.

[CR86] Panos SE, Hinkin CH, Singer EJ, Thames AD, Patel SM, Sinsheimer JS, Del Re AC, Gelman BB, Morgello S, Moore DJ, Levine AJ (2013). Apolipoprotein-E genotype and human immunodeficiency virus-associated neurocognitive disorder: the modulating effects of older age and disease severity. Neurobehav HIV Med.

[CR87] Pathai S, Lawn SD, Gilbert CE, McGuinness D, McGlynn L, Weiss HA, Port J, Christ T, Barclay K, Wood R, Bekker LG, Shiels PG (2013). Accelerated biological ageing in HIV-infected individuals in South Africa: a case-control study. AIDS.

[CR88] Pathai S, Bajillan H, Landay AL, High KP (2014). Is HIV a model of accelerated or accentuated aging?. J Gerontol A Biol Sci Med Sci.

[CR89] Peluso R, Haase A, Stowring L, Edwards M, Ventura P (1985). A Trojan horse mechanism for the spread of visna virus in monocytes. Virology.

[CR90] Pulliam L (2009). HIV regulation of amyloid beta production. J NeuroImmune Pharmacol.

[CR91] Qiu Z, Hyman BT, Rebeck GW (2004). Apolipoprotein E receptors mediate neurite outgrowth through activation of p44/42 mitogen-activated protein kinase in primary neurons. J Biol Chem.

[CR92] Rahimian P, He JJ (2016). HIV-1 Tat-shortened neurite outgrowth through regulation of microRNA-132 and its target gene expression. J Neuroinflammation.

[CR93] Rea IM, Mc Dowell I, McMaster D, Smye M, Stout R, Evans A (2001). Apolipoprotein E alleles in nonagenarian subjects in the Belfast Elderly Longitudinal Free-living Ageing Study (BELFAST). Mech Ageing Dev.

[CR94] Reddy PV, Gandhi N, Samikkannu T, Saiyed Z, Agudelo M, Yndart A, Khatavkar P, Nair MP (2012). HIV-1 gp120 induces antioxidant response element-mediated expression in primary astrocytes: role in HIV associated neurocognitive disorder. Neurochem Int.

[CR95] Rickabaugh TM, Baxter RM, Sehl M, Sinsheimer JS, Hultin PM, Hultin LE, Quach A, Martinez-Maza O, Horvath S, Vilain E, Jamieson BD (2015). Acceleration of age-associated methylation patterns in HIV-1-infected adults. PLoS One.

[CR96] Roberts RO, Cha RH, Mielke MM, Geda YE, Boeve BF, Machulda MM, Knopman DS, Petersen RC (2015). Risk and protective factors for cognitive impairment in persons aged 85 years and older. Neurology.

[CR97] Sabatier JM, Vives E, Mabrouk K, Benjouad A, Rochat H, Duval A, Hue B, Bahraoui E (1991). Evidence for neurotoxic activity of tat from human immunodeficiency virus type 1. J Virol.

[CR98] Sabo T, Lomnitski L, Nyska A, Beni S, Maronpot RR, Shohami E, Roses AD, Michaelson DM (2000). Susceptibility of transgenic mice expressing human apolipoprotein E to closed head injury: the allele E3 is neuroprotective whereas E4 increases fatalities. Neuroscience.

[CR99] Sacktor N. (2018). Amyloid uptake by PET imaging suggest premature aging in older HIV+ individuals. Themed Discussion: Aging Brain and HIV: Before and After. Conference on Retroviruses and Opportunistic Infections; March 6, 2018; Boston, MA. http://www.croiwebcasts.org

[CR100] Sacktor N, Skolasky R, Selnes OA, Watters M, Poff P, Shiramizu B, Shikuma C, Valcour V (2007). Neuropsychological test profile differences between young and old human immunodeficiency virus-positive individuals. J Neurovirol.

[CR101] Sacktor N, Skolasky RL, Seaberg E, Munro C, Becker JT, Martin E, Ragin A, Levine A, Miller E (2016). Prevalence of HIV-associated neurocognitive disorders in the Multicenter AIDS Cohort Study. Neurology.

[CR102] Schachter F, Faure-Delanef L, Guenot F, Rouger H, Froguel P, Lesueur-Ginot L, Cohen D (1994). Genetic associations with human longevity at the APOE and ACE loci. Nat Genet.

[CR103] Schouten J, Wit FW, Stolte IG, Kootstra NA, van der Valk M, Geerlings SE, Prins M, Reiss P (2014). Cross-sectional comparison of the prevalence of age-associated comorbidities and their risk factors between HIV-infected and uninfected individuals: the AGEhIV cohort study. Clin Infect Dis.

[CR104] Scott JC, Woods SP, Carey CL, Weber E, Bondi MW, Grant I (2011). Neurocognitive consequences of HIV infection in older adults: an evaluation of the “cortical” hypothesis. AIDS Behav.

[CR105] Sheppard DP, Woods SP, Bondi MW, Gilbert PE, Massman PJ, Doyle KL (2015). Does older age confer an increased risk of incident neurocognitive disorders among persons living with HIV disease?. Clin Neuropsychol.

[CR106] Sheppard DP, Iudicello JE, Morgan EE, Kamat R, Clark LR, Avci G, Bondi MW, Woods SP (2017). Accelerated and accentuated neurocognitive aging in HIV infection. J Neurovirol.

[CR107] Simioni S, Cavassini M, Annoni JM, Rimbault Abraham A, Bourquin I, Schiffer V, Calmy A, Chave JP, Giacobini E, Hirschel B, Du Pasquier RA (2010). Cognitive dysfunction in HIV patients despite long-standing suppression of viremia. AIDS.

[CR108] Solomon A, Tennakoon S, Leeansyah E, Arribas J, Hill A, Van Delft Y, Moecklinghoff C, Lewin SR (2014). No difference in the rate of change in telomere length or telomerase activity in HIV-infected patients after three years of darunavir/ritonavir with and without nucleoside analogues in the MONET trial. PLoS One.

[CR109] Soontornniyomkij V, Moore DJ, Gouaux B, Soontornniyomkij B, Tatro ET, Umlauf A, Masliah E, Levine AJ, Singer EJ, Vinters HV, Gelman BB, Morgello S, Cherner M, Grant I, Achim CL (2012). Cerebral beta-amyloid deposition predicts HIV-associated neurocognitive disorders in APOE epsilon4 carriers. AIDS.

[CR110] Strain JF, Burdo TH, Song SK, Sun P, El-Ghazzawy O, Nelson B, Westerhaus E, Baker L, Vaida F, Ances BM (2017). Diffusion basis spectral imaging detects ongoing brain inflammation in virologically well-controlled HIV+ patients. J Acquir Immune Defic Syndr.

[CR111] Theendakara V, Peters-Libeu CA, Spilman P, Poksay KS, Bredesen DE, Rao RV (2016). Direct transcriptional effects of apolipoprotein E. J Neurosci.

[CR112] Thompson KA, Cherry CL, Bell JE, McLean CA (2011). Brain cell reservoirs of latent virus in presymptomatic HIV-infected individuals. Am J Pathol.

[CR113] Triant VA (2012). HIV infection and coronary heart disease: an intersection of epidemics. J Infect Dis.

[CR114] Triant VA (2014). Epidemiology of coronary heart disease in patients with human immunodeficiency virus. Rev Cardiovasc Med.

[CR115] Triant VA, Lee H, Hadigan C, Grinspoon SK (2007). Increased acute myocardial infarction rates and cardiovascular risk factors among patients with human immunodeficiency virus disease. J Clin Endocrinol Metab.

[CR116] Tuminello Elizabeth R., Han S. Duke (2011). The Apolipoprotein E Antagonistic Pleiotropy Hypothesis: Review and Recommendations. International Journal of Alzheimer's Disease.

[CR117] Turana Y, Suzy Handajani Y, Widjaja NT (2015). Association between APOE epsilon4 genotype and memory impairment in elderly with normal global cognitive assessment. Diagnostics (Basel).

[CR118] Turchan-Cholewo J, Liu Y, Gartner S, Reid R, Jie C, Peng X, Chen KC, Chauhan A, Haughey N, Cutler R, Mattson MP, Pardo C, Conant K, Sacktor N, McArthur JC, Hauser KF, Gairola C, Nath A (2006). Increased vulnerability of ApoE4 neurons to HIV proteins and opiates: protection by diosgenin and L-deprenyl. Neurobiol Dis.

[CR119] Valcour V, Shikuma C, Shiramizu B, Watters M, Poff P, Selnes O, Holck P, Grove J, Sacktor N (2004). Higher frequency of dementia in older HIV-1 individuals: the Hawaii Aging with HIV-1 Cohort. Neurology.

[CR120] Valcour V, Shikuma C, Shiramizu B, Watters M, Poff P, Selnes OA, Grove J, Liu Y, Abdul-Majid KB, Gartner S, Sacktor N (2004). Age, apolipoprotein E4, and the risk of HIV dementia: the Hawaii Aging with HIV Cohort. J Neuroimmunol.

[CR121] Valcour V, Paul R, Neuhaus J, Shikuma C (2011). The effects of age and HIV on neuropsychological performance. J Int Neuropsychol Soc.

[CR122] Valcour V, Chalermchai T, Sailasuta N, Marovich M, Lerdlum S, Suttichom D, Suwanwela NC, Jagodzinski L, Michael N, Spudich S, van Griensven F, de Souza M, Kim J, Ananworanich J (2012). Central nervous system viral invasion and inflammation during acute HIV infection. J Infect Dis.

[CR123] Vitek MP, Brown CM, Colton CA (2009). APOE genotype-specific differences in the innate immune response. Neurobiol Aging.

[CR124] Wendelken LA, Valcour V (2012). Impact of HIV and aging on neuropsychological function. J Neurovirol.

[CR125] Wendelken LA, Jahanshad N, Rosen HJ, Busovaca E, Allen I, Coppola G, Adams C, Rankin KP, Milanini B, Clifford K, Wojta K, Nir TM, Gutman BA, Thompson PM, Valcour V (2016). ApoE epsilon4 is associated with cognition, brain integrity, and atrophy in HIV over age 60. J Acquir Immune Defic Syndr.

[CR126] Wenzel ED, Bachis A, Avdoshina V, Taraballi F, Tasciotti E, Mocchetti I (2017). Endocytic trafficking of HIV gp120 is mediated by dynamin and plays a role in gp120 neurotoxicity. J NeuroImmune Pharmacol.

[CR127] Woods SP, Dawson MS, Weber E, Grant I (2010). The semantic relatedness of cue-intention pairings influences event-based prospective memory failures in older adults with HIV infection. J Clin Exp Neuropsychol.

[CR128] Wu B, Huang Y, Braun AL, Tong Z, Zhao R, Li Y, Liu F, Zheng JC (2015). Glutaminase-containing microvesicles from HIV-1-infected macrophages and immune-activated microglia induce neurotoxicity. Mol Neurodegener.

[CR129] Xu PT, Schmechel D, Rothrock-Christian T, Burkhart DS, Qiu HL, Popko B, Sullivan P, Maeda N, Saunders AM, Roses AD, Gilbert JR (1996). Human apolipoprotein E2, E3, and E4 isoform-specific transgenic mice: human-like pattern of glial and neuronal immunoreactivity in central nervous system not observed in wild-type mice. Neurobiol Dis.

[CR130] Yamamoto T, Choi HW, Ryan RO (2008). Apolipoprotein E isoform-specific binding to the low-density lipoprotein receptor. Anal Biochem.

[CR131] Zanet DL, Thorne A, Singer J, Maan EJ, Sattha B, Le Campion A, Soudeyns H, Pick N, Murray M, Money DM, Cote HC (2014). Association between short leukocyte telomere length and HIV infection in a cohort study: no evidence of a relationship with antiretroviral therapy. Clin Infect Dis.

[CR132] Zenon F, Cantres-Rosario Y, Adiga R, Gonzalez M, Rodriguez-Franco E, Langford D, Melendez LM (2015). HIV-infected microglia mediate cathepsin B-induced neurotoxicity. J Neurovirol.

[CR133] Zhou Y, Liu J, Xiong H (2017). HIV-1 glycoprotein 120 enhancement of N-methyl-D-aspartate NMDA receptor-mediated excitatory postsynaptic currents: implications for HIV-1-associated neural injury. J NeuroImmune Pharmacol.

