# Supplementary material for: Aging and Apolipoprotein E in HIV Infection
Source: J Neurovirol. 2018 Jul 9;24(5):529–48. doi: 10.1007/s13365-018-0660-2 (PMC6244718; doi:10.1007/s13365-018-0660-2)
Supplement: Supplementary file 1 — (DOC 85 kb) [file 13365_2018_660_MOESM1_ESM.doc]

Table S1 Animal and in vitro Studies of ApoE Effects on the CNS

| Publication | Experimental system | Measures | Findings |
| --- | --- | --- | --- |
| Turchan-Cholewo | Neuronal cultures prepared from fetal brains and genotyped for ApoE alleles | Neurotoxicity as measured by changes in mitochondrial membrane potential | Neurotoxicity was maximal in Apoε4/ε4 neurons when exposed to HIV gp120 and Tat, and in Apoε3/ε4 or Apoε4/ε4 neurons exposed to morphine and Tat, as compared to neurons harboring any other genotype. |
| Maezawa | Microglia or astrocytes derived from human ApoE2, ApoE3 and ApoE4 targeted replacement (TR) mice cocultured with neurons and activated with LPS | Neurotoxicity as measured by NeuN-immunoreactive cells. | Neurotoxicity was greater in microglia than astrocytes when treated with LPS.  ApoE4 microglia were more neurotoxic than ApoE3  Microglia secreted IL-6 and TNFα levels as: TR APOE4 > TR APOE3 > TR APOE2 |
| Maezawa | Human ApoE2, ApoE3 and ApoE4 TR mice | Dendrite length | After intracerebroventricular LPS injection, dendrite length decrease was the same for all isoforms, but dendrites in ApoE4 TR failed to recover |
| Maezawa | Primary astrocyte cultures from human ApoE2, ApoE3 and ApoE4 TR mice stimulated with LPS | Cytokine secretion | IL-6, TNFα and IL-1β were secreted in larger amounts from astrocytes derived from ApoE3 TR mice than ApoE4 |
| Burt | HIV susceptible cells MAGI R5 or SupT1 CCR5 infected with pseudotyped HIV in the presence of ApoE proteins | Fluorescence measures | Addition of ApoE4 to the medium increases HIV fusion and HIV entry into target cells as compared to ApoE3 |
| Vitek | Microglia and peripheral macrophage derived from human ApoE2, ApoE3 and ApoE4 TR mice | Release of cytokines after stimulation with polycytidylic: polyinosinic acid (PIC) or LPS and/or interferon gamma (IFNγ) | Release of nitric oxide (NO) and cytokines after activation with PIC or LPS were higher in microglia cultures from ApoE4 TR mice than ApoE3 TR mice. Macrophages from ApoE4 TR mice released more NO after activation with PIC + IFN γ or LPS + IFNγ than ApoE3 TR mice |
| Dumanis | Human ApoE2, ApoE3 and ApoE4 TR mice  Primary cortical neurons in culture treated with recombinant ApoE | Golgi staining and dendrite morphology | ApoE3 increases and ApoE4 decreases neuronal spine density  Dendritic spines were shorter in ApoE4 TR mice than ApoE3 TR mice |
| Crawford | Controlled cortical impact model of brain injury (TBI) evaluated in transgenic mice heterozygous for ApoE isoforms | Gene expression in the hippocampus and cortex | A larger number of genes are differentially regulated after TBI in ApoE3 mice that in ApoE4 mice.  Differentially expressed gene functions include cellular growth and differentiation, cellular movement and immunological disease, among others. |
| Theendekara | Human neuroblastoma and glioblastoma  Skin fibroblasts from patients with Alzheimer’s disease  ApoE TR mouse brains | Chromatin immunoprecipitation and high-throughput DNA sequencing | Transfected ApoE can translocate to the nucleus and is capable of high affinity binding to DNA, including binding to 1700 promoter regions, presumably modulating their expression.  Functions for the genes expressed from these promoters include important functions associated with Alzheimer’s disease |
| Geffin | Human neuronal progenitor cell line hNP1 (endogenous Apoε3/ε3 genotype) exposed to HIV and to recombinant ApoE3 or ApoE4 | Gene expression (microarray) | Addition of rApoE4 resulted in the downregulation more genes as compared to rApoE3.  Half of 85 ApoE4-downregulated genes involved processes associated with neurogenesis.  Downregulation effect magnified in cells exposed to HIV |
